# Engineering with EP2/EP4 knockout and IL-15 transpresentation renders stem cell-derived NK cells self-persistent and resistant to PGE2 inhibition

**DOI:** 10.3389/fimmu.2026.1792303

**Published:** 2026-06-26

**Authors:** Marcos Vidal-Manrique, Laura Hooijmaijers, Julia A.J. Teders, Elif Saf, Kjell Klarenbeek, Iris M. Hagemans, Jorge Cuenca-Escalona, Alessandra Cambi, I. Jolanda M. de Vries, Paul K. J. D. de Jonge, Anniek B. van der Waart, Harry Dolstra

**Affiliations:** 1 Department of Laboratory Medicine, Laboratory of Hematology, Radboud university medical center, Nijmegen, Netherlands; 2 Department of Medical BioSciences, Radboud university medical center, Nijmegen, Netherlands

**Keywords:** adoptive cell transfer, CRISPR, hematopoietic stem cells, IL-15 transpresentation, NK cells, prostaglandin E2

## Abstract

Adoptive Natural Killer (NK) cell transfer is a promising therapy for the treatment of cancer. We developed a GMP-compliant protocol to generate NK cells from hematopoietic stem and progenitor cells (HSPC-NK). However, the immunosuppressive tumor microenvironment — especially prostaglandin E2 (PGE2) signaling via E-prostanoid receptors EP2 and EP4— and lack of persistence of allogeneic NK cells hinders HSPC-NK therapeutic efficacy. Here, we enhanced the therapeutic efficacy of HSPC-NK cells by interfering with both EP2 and EP4 with antagonists or CRISPR-KO, and through engineering with IL-15 tethered to membrane-bound IL-15Rα (tIL15). We found that CRISPR interference of EP2 and EP4 fully rescued proliferation and potency of HSPC-NK cells under PGE2 rich conditions. In addition, combined EP2/EP4 KO and tIL15 engineering renders HSPC-NK cells resistant to PGE2, while improving their survival and functionality against different tumor targets in cytokine-deprived environments. Altogether, our EP2/EP4-KO tIL15-HSPC-NK cells pose a promising therapy for cancer.

## 1 Introduction

Natural Killer (NK) cells inherently recognize and elicit cytotoxic responses against tumor-transformed cells through a balance of inhibitory and activating receptors. Given their natural anti-tumor potential and their suitability for allogeneic use, adoptive transfer of NK cells has become an alternative attractive therapy for cancer treatment (1). In fact, many clinical trials have positively associated high NK cell frequencies with improved survival in both hematological and solid malignancies (2–4), which highlights the beneficial effect of NK cells in tumor growth control. In this context, we have developed a GMP-compliant, cytokine-based, feeder-free protocol for the generation of billions of NK cells from hematopoietic stem and progenitor cells obtained from donor umbilical cord blood (HSPC-NK) (5). Importantly, our HSPC-NK cells have been clinically investigated for the treatment of acute myeloid leukemia (NCT04347616) (5) and ovarian cancer (NCT03539406) (6), in which they were safely administered without inducing severe toxicity (7). However, although transient clinical responses have been observed in NK cell trials, improvements are needed to induce more long-lasting and curative responses (8, 9), underscoring the need to further optimize NK cell immunotherapies for cancer treatment.

Full clinical efficacy of allogeneic NK cells for cancer treatment is hampered by the strong immune suppressive microenvironment (10), as well as the insufficient survival and expansion of NK cells upon infusion (11). The suppression of NK cells could be tackled by targeting key inhibitory factors from the tumor microenvironment. In this context, multiple studies have substantiated that increased levels of prostaglandin E2 (PGE2), or the upregulation of the PGE2-producing enzymes cyclooxygenase type 1 and 2 (COX-1/2), associate with worse prognosis in multiple malignancies, including gliomas (12), colorectal cancer (13, 14), breast cancer (15, 16), lung cancer (17), head and neck cancer (18), gastric cancer (19), pancreatic cancer (20), cervical cancer (21), ovarian cancer (22–24), esophageal squamous cell carcinoma (25, 26), hepatocellular carcinoma (27) and melanoma (28, 29). For this reason, we and others have targeted the surface receptors interacting with PGE2 that drive suppression of anti-tumor immune responses in humans, namely E prostanoid receptor 2 (EP2) and 4 (EP4) (30–33). Importantly, PGE2 has also been shown to inhibit the anti-tumor functionality of human NK cells by inducing their apoptosis, reducing their tumor migration and infiltration, and inhibiting cytokine production and cytotoxic responses (34–37). However, it is still unknown whether our HSPC-NK cell products negatively respond to PGE2 stimulation, and whether targeting of PGE2-EP2/EP4 axis could improve their anti-tumor responses. Furthermore, the survival and expansion of HSPC-NK cells can be improved through cytokine stimulation. Importantly, we and others have substantiated that (HSPC-)NK cells’ expansion can be enhanced via the IL-15 transpresentation (tIL15) mechanism, which results from the co-expression of IL-15 cytokine and its membrane-bound receptor alpha component (IL-15Rα) (38, 39). However, superior HSPC-NK cell proliferation was observed upon co-culture with tumor cells transiently transfected with tIL15-encoding mRNA (38). Alternatively, long-term survival and expansion could be more stable with direct, constitutive engineering of HSPC-NK cells with tIL15.

In this study, we investigated the expression of EP2 and EP4 in our HSPC-NK cells and exploited different strategies to counteract PGE2-mediated suppression. In addition, we studied whether tIL15 engineering enhanced survival and expansion of HSPC-NK cells. Based on our results, we anticipate that multi-engineering of HSPC-NK cells with EP2- and EP4-knockout and tIL15 expression could significantly improve their therapeutic effect for the treatment of cancer.

## 2 Materials and methods

### 2.1 Cell lines and culture

K562 (ATCC) and Jurkat (ATCC) cells were cultured in IMDM media (Gibco, Cat# 21980-32) supplemented with 10% heat-inactivated (HI) FBS (Corning, Cat# 35079004), and split twice per week at 0.1*10^6^ cells/mL. KHYG-1 cells kindly provided by Stikvoort A., et al. (40) were cultured in DMEM-Glutamax (Gibco, Cat# 31966-021) supplemented with 10% HI-FBS and 1000 IU/mL IL-2 (Proleukin, Chiron, Cat# NDC 53905-991-01) and split thrice per week at 0.5-0.75*10^6^ cells/mL. Ovarian cancer cells SKOV-3 (ATCC) were cultured in RPMI 1640 (Gibco, Cat# 11875093) supplemented with 10% HI-FBS. MCF-7 (ATCC) was cultured in IMDM supplemented with 10% FCS. A375, provided by Cuenca-Escalona J., et al. (30), and PhoenixAMPHO cells (ATCC) were cultured in DMEM supplemented with 10% HI-FBS, and 2mM glutamine (Phoenix AMPHO). All adherent cell lines were split twice per week through PBS washing and subsequent trypsinization (Gibco, Cat# 25300054). All cells were cultured at 37 °C and 5% CO_2_. All cultures tested negative for MycoAlert^®^ PLUS Mycoplasma Detection Kit (Lonza, Cat# LT07-710).

### 2.2 HSPC-NK cell generation and culture

Umbilical cord blood was collected in cesarean sections after informed consent (CMO 2014/226), in accordance with the Declaration of Helsinki. Cord blood-derived stem cells were enriched with CliniMACS^®^ CD34 Reagent CR/GMP beads (Miltenyi Biotec, Cat# 170-076-711) and differentiated into HSPC-NK cells as previously described (41). HSPC-NK cells (all comprising ≥92% CD56^+^ cells) were either directly used for functional assays or cryopreserved. When needed, HSPC-NK cells were thawed and cultured for a period of 5–9 days in NK MACS Basal medium containing 1:100 NK MACS supplement (Miltenyi Biotec, Cat# 130-114-429), 10% pooled human serum (Sanquin), 50ng/mL IL-15 (ImmunoTools, Cat# 11340157) and 0.2ng/mL IL-12 (ImmunoTools, Cat# 11349127), before using them for functional assays.

### 2.3 Retrovirus production and transduction

Retroviruses were harvested from PhoenixAMPHO cells two days after plasmid transfection with calcium phosphate. For the transduction of Jurkats for retroviral titer determination or day 3 and 7-expanded CD34^+^ stem cells, retronectin-coated plates (Takara, Cat# T100B) were used following manufacturer’s indications. In short, 24- and 6-well plates were coated with retronectin for 24h overnight or 2h at room temperature before washing with PBS and adding the desired amount of virus for centrifugation at 4500rpm for 1.5h at 4 degrees. After removal of unbound retrovirus, a total of 0.3*10^6^ Jurkat cells in 24-well plate or 0.15-0.3*10^6^ or 0.4-0.6*10^6^ stem cells in 24 and 6-well plates, respectively, were plated and incubated for 3–4 days.

### 2.4 CRISPR knockout

For the preparation of the RNP complex, Atto550-labeled tracrRNA (IDT, Cat# 1075927) and target-specific crRNA (IDT, **Supplementary Table 1**) were first annealed at 1:1 ratio for 5 minutes at 95 °C and subsequently cooled down for 20 minutes at room temperature (RT). The resulting guide RNA (gRNA) was mixed with Cas9 enzyme (IDT, Cat# 1081062) at a 1:2.4 Cas9:gRNA molar ratio to generate the RNP complex. After 20 minutes, the RNP complex was mixed with an electroporation enhancer (IDT, Cat# 1075916) at a final concentration of 4μM.

0.5-2.5*10^6^ differentiating HSPC-NK cells from day 28, or CD34^+^ stem cells from days 3 or 5 of the expansion/differentiation protocol, were thoroughly washed with PBS to remove culture components that could hamper nucleofection efficacy. Cells were then resuspended in in-house-prepared mannitol buffer (5 mM KCl, 15 mM MgCl_2_, 15 mM HEPES, 150 mM phosphate buffer, 50 mM mannitol; also known as Sol2 buffer) and finally combined with or without the RNP complexes to a final volume of 21μL. Nucleofection was performed using Lonza 4D nucleofector (AAF-1003X) using CM-137 pulse code for d28 HSPC-NK cells, or EO-100 pulse code for day 3/5-expanded stem cells. Electroporation efficiency was measured by quantifying Atto550 on flow cytometry (see below).

### 2.5 Flow cytometry analysis

Viability dyes used include Sytox Blue (ThermoFisher Scientific, Cat# S34857), 7-AAD (Sigma-Aldrich, Cat# A9400) and Fixable Viability Dye eFluor780 (Invitrogen, Cat# 65-0865-14). Cells were resuspended in Fc-blocking buffer containing human immunoglobulins (Sanquin, Cat# 0041-100) 50μg/mL in FACS buffer (PBS containing 0.5% BSA (Sigma, Cat# 9048-46-8)) before staining with antibodies pre-titrated in PBMCs or HSPC-NK cells. For intracellular cytokine measurement, cells were permeabilized according to the fixation/permeabilization kit (Invitrogen, Cat# 00-5523) and stained with antibodies in permeabilization buffer. All antbodies used for flow cytometry analysis are included in **Supplementary Table 2**. All cells were resuspended in FACS containing 1% PFA, when needed, and measured on 13-color Cytoflex or 21-color Cytoflex LX (Backman Coulter), and analyzed with Kaluza 2.3 (Beckman Coulter).

### 2.6 Functional readouts

#### 2.6.1 Proliferation assay

Fresh HSPC-NK cells were labeled with 10 µM Cell Proliferation Dye eFluor450 (Invitrogen, Cat# 65-0842-85) following manufacturer’s indications for 10 minutes at 37 °C. Unbound dye was quenched with IMDM containing 10% HI-FBS (1:1 v/v) for 5 minutes at 4 °C. 1*10^6^ cells/mL eFluor450-labeled HSPC-NK cells/mL were cultured in NK MACS medium + 10% HS containing 20ng/mL of IL-15 in the presence or absence of indicated concentrations of PGE2 (Prostin, Cat# G02AD02) and EP2 antagonist AH6809 (Cayman Chemical, Cat# 33458-93-4) and EP4 antagonist L-161982 (Cayman Chemical, Cat# 147776-06-5) for up to 4–6 days. Proliferation score was calculated as fold reduction of eFluor450 median fluorescence intensity (MFI) as: (1/efluor450 MFI of life CD56^+^ cells after 4–6 days)/(1/efluor450 MFI of life CD56^+^ cells on day zero of plating). Fold expansion was calculated comparing the number of life CD56^+^ cells on day 4–6 vs. day zero.

#### 2.6.2 Degranulation and potency assay

1*10^6^ HSPC-NK cells/mL were cultured with or without 20 ng/mL of IL-15 in the presence or absence of 100nM PGE2 and EP2 and EP4 antagonists for 20h. Next, HSPC-NK cells were washed twice with PBS to eliminate any cytokines and factors derived from the pre-stimulation that might confound the functional readout. Finally, HSPC-NK cells were plated in fresh IMDM + 10% HI-FBS with the indicated targets at an effector-to-target ratio (E:T) of 1.5:1, with addition of CD107a antibody (**Supplementary Table 2**). 1 h after plating, BD GolgiPlug (BD Biosciences, Cat# 555029) was added. After 4 h incubation, cells were permeabilized and stained for intracellular cytokines as indicated below.

#### 2.6.3 Killing assay

HSPC-NK cells were labeled with eFluor450 as previously indicated. Then 1*10^6 HSPC-NK cells/mL were cultured in IMDM + 10% FBS the presence or absence of 1000nM PGE2 for 40h with the indicated targets at a 0.5:1 E:T ratio. The number of viable target cells was measured by flow cytometry as 7-AAD^–^eFluor450^–^. Percentage of killing was calculated as follows: 1 – ((number of target viable cells in co-culture with HSPC-NK cells)/(number of viable target cells cultured alone)) * 100.

### 2.7 Cytokine production and ELISA

1*10^6^ HSPC-NK cells/mL were cultured in 20ng/mL of IL-15 in the presence or absence of 1-1000nM PGE2 for 24h. For targets, cells were plated regularly (control), or stimulated with 10ng/mL IFNγ (ImmunoTools, Cat# 11343536) or IL-6 (ImmunoTools, Cat# 11340066) for three days, after which supernatant of cells was kept in -20 until use. Culture supernatants were later thawed and evaluated for IFNγ (Invitrogen, Cat# 88-7316) TNF (Mabtech, Cat# 3512-1H) or PGE2 (ThermoFisher Scientific, Cat# EHPGE2), following manufacturer’s instructions. H_3_PO_4_ was used to stop the coloration of samples at the end of the assay. Differences in optical density (OD) were measured with FlexStation 3 Multi-Mode Microplate Reader (Molecular Devices).

### 2.8 DNA/RNA isolation and quantitative PCR

DNA and RNA were extracted following the instructions of NucleoSpin Blood QuickPure kit (Macherey-Nagel, Cat# 740569.250) and NucleoSpin RNA kit (Macherey-Nagel, Cat# 740955.250), respectively. cDNA conversion from RNA was performed using Moloney Murine Leukemia Virus (M-MLV) Reverse Transcriptase (RT) (Invitrogen, Cat# 28-025-013) according to the manufacturer’s instructions in Veriti Thermal Cycler (Applied Biosystems). Quantitative PCR (qPCR) from 25-50ng of genomic DNA or 12.5ng of cDNA was performed using SYBR Green Universal Master Mix (Life Technologies, Cat# 4309155) and measured in 7500 Real Time PCR System (Applied Biosystems). For transcript quantification, human GAPDH TaqMan probe (Applied Biosystems, Cat# 402869) was used to normalize expression across samples. The percentage of PTGER2/4 mutations at the transcript level or in the genomic DNA was determined through getPCR technique (42). The list of primers used for qPCR and getPCR, as well as detailed information on their usage, is shown in **Supplementary Table 3**. Amplicon sizes were validated in agarose gel (Invitrogen, Cat# 15510-027) containing Ethidium Bromide and measured in GelDoc Go Imaging System (Bio-Rad).

### 2.9 CRISPR editing analysis

Genomic regions flanking the PTGER2 and PTGER4 cut sites targeted by each gRNA were amplified through PCR from 100ng of genomic DNA using the Phusion high-fidelity DNA polymerase (New England Biolabs, Cat# E0553S). **Supplementary Table 3** shows the primers flanking each gRNA cut site. The resulting amplicons were subjected to PCR Cleanup using NucleoSpin Gel and PCR Clean-up Kit (Macherey-Nagel, Cat# 740609.50) according to the manufacturer’s instructions and sequenced by the Radboudumc Sequencing Facility. Quantification of percentage of indels and KO was determined using the online analysis tool Inference of CRISPR Edits (ICE, Synthego).

### 2.10 Quantification and statistical analysis

Statistical analysis was performed using Graphpad Prism v.10.4.1. Error bars show SEM or SD when data come from independent donors or replicates, respectively. Whenever indicated in the figure captions, paired parametric Student *t* tests or non-parametric Wilcoxon tests were used for pairwise comparisons. Non-parametric Kruskal-Wallis or parametric One-way ANOVA and two-way ANOVAs were used when indicated with Tukey, Sidak or Dunnett corrections when recommended by the software. Significance was defined as *p* < 0.05 (*), *p* < 0.01 (**), *p* < 0.001 (***) and *p* < 0.0001 (****).

## 3 Results

### 3.1 HSPC-NK cells express EP2 and EP4 and are negatively affected by PGE2

**Figure 1:**
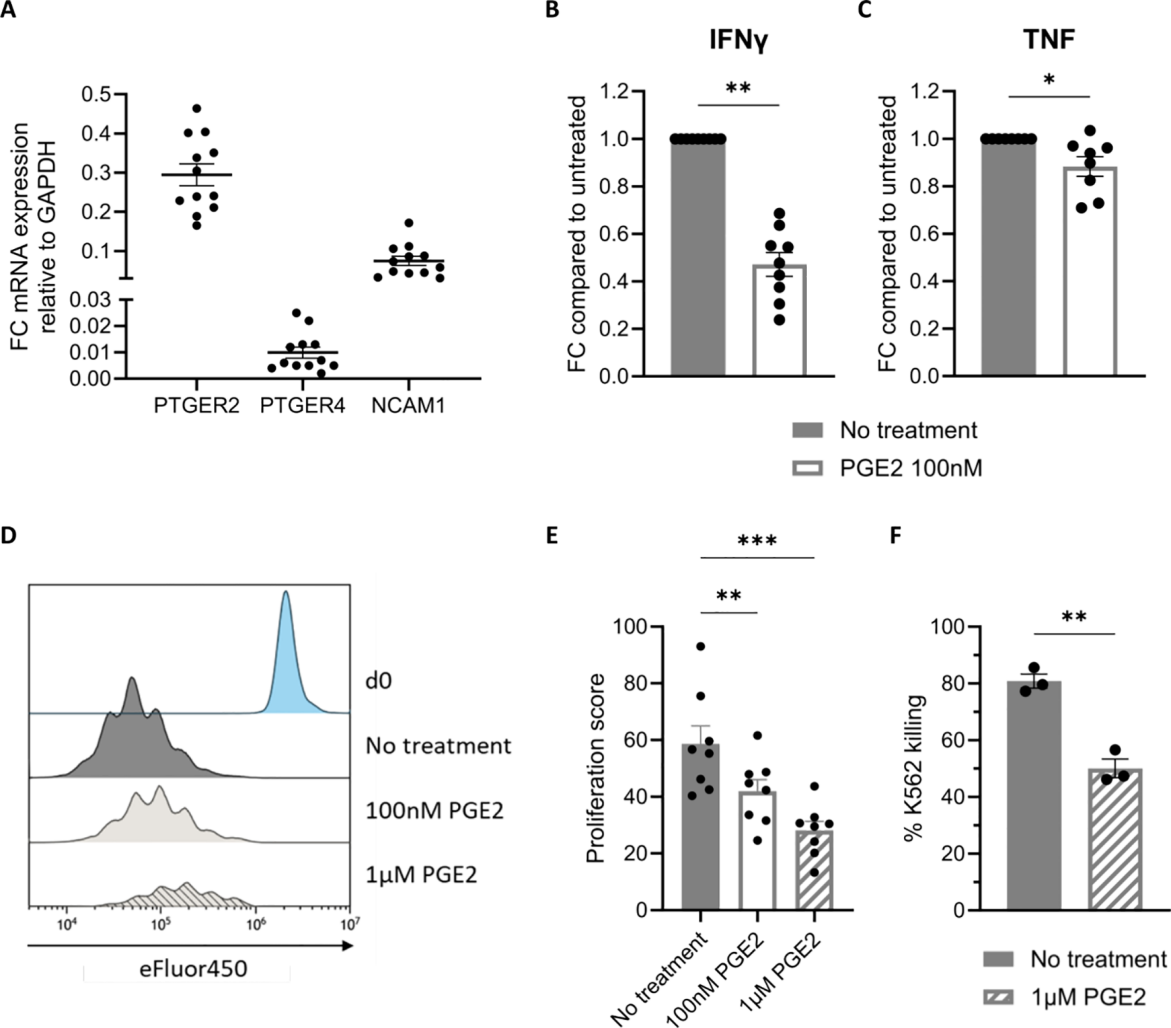
HSPC-NK cells express PTGER2 and 4 and are inhibited by PGE2. **(A)** Expression of PTGER2, PTGER4 and NCAM1 on cDNA relative to GAPDH from differentiated HSPC-NK cells (N = 12). Line and error bars indicate mean ± SEM, respectively. **(B)** IFNγ (N = 9) and **(C)** TNF (N = 8) secretion of 20ng/mL IL-15-stimulated HSPC-NK cells treated (or not) with 100nM PGE2 for 24h relative to non-PGE2 treated HSPC-NK cells. Bars and error bars indicate mean ± SEM, respectively. **(D)** Representative flow cytometry histograms showing the different degrees of eFluor450 dye dilution upon HSPC-NK cell divisions stimulated with 20ng/mL IL-15 and treated with (or without) different PGE2 concentrations. **(E)** Proliferation of 20ng/mL IL-15-stimulated HSPC-NK cells (N = 8) treated (or not) with either 100nM or 1μM of PGE2 for six days, and measured by the fold decrease in the MFI of the proliferation dye eFluor450 compared to plating day 0. Bars and error bars indicate mean + SEM, respectively. **(F)** K562 killing by HSPC-NK cells after co-culture for 40h containing (or not) 1μM PGE2. Bars and error bars indicate mean ± SEM, respectively. ns, no significance; **p < 0.01, ***p < 0.001; **(B, C)** Paired Wilcoxon test, **(E)** One-way ANOVA with Dunnett correction, **(F)** Paired t-test.

PGE2 binds four EP receptors (EP1-4) on the surface of human cells. NK cells express EP2, EP3 and EP4 but not EP1 (43). However, whether our HSPC-NK cells show similar EP receptor expression patterns remains elusive. Therefore, we first sought to determine the expression of EP receptors on HSPC-NK cells. HSPC-NK cells showed higher expression of *PTGER2*, and lower expression of *PTGER4*, compared to the common NK cell marker *NCAM1* (**Figure 1A**), whereas PTGER1 and PTGER3 expression was undetectable by qPCR, validating the phenotype previously reported in other human NK cell sources (35).

Since both EP2 and EP4 have been reported to mediate NK cell suppression by PGE2 (35, 44), we next sought to study whether PGE2 suppressed the functionality of HSPC-NK cells. For this, we first stimulated HSPC-NK cells with IL-15 in the presence or absence different PGE2 concentrations (**Supplementary Figure 1A**). After 24h, we observed that ≥25nM PGE2 induced a significant decrease in IFNγ (**Supplementary Figure 1B**). Following these results, and in concomitance with the doses used in other reports assessing the *in vitro* functionality of human NK cells (37, 43), ≥100nM PGE2 was selected for further functional evaluation. Importantly, PGE2 decreased secretion of IFNγ and TNF without compromising HSPC-NK cell viability (**Figures 1B, C**; **Supplementary Figures 1C–E**). In addition, we observed a significant decrease in the proliferative capacity of HSPC-NK cells with increased concentrations of PGE2 after six days (**Figures 1D, E**). Furthermore, addition of PGE2 significantly decreased HSPC-NK cytotoxic activity against K562 after 40h with no detectable differences in HSPC-NK cell numbers (**Figure 1F**, **Supplementary Figure 1F**). On the other hand, the phenotype of IL-15-stimulated HSPC-NK cells was only modestly influenced by PGE2 after 6 days, with changes predominantly confined to the degree of expression of activating receptors (**Supplementary Figures 1G, H**). Overall, these data indicate that our HSPC-NK cells are susceptible to suppression by the PGE2-EP2/EP4 axis.

### 3.2 EP2 and EP4 antagonists rescue cytokine production, but not proliferation, of PGE2-suppressed HSPC-NK cells in the short term

As tumor cells are known to produce PGE2 ***in vivo***, we first studied whether PGE2 could be secreted by tumor cells ***in vitro***. Here, we studied K562 cells as a hematological malignancy model and different ovarian cancer cells as solid tumor models, as well as HSPC-NK cells themselves. No PGE2 production was observed by either cell when compared to their respective serum-containing medium only, even when stimulated with IFNγ or IL-6 (**Supplementary Figures 2A–C**). Therefore, to mimic PGE2-mediated suppression in ***in vitro*** co-culture assays with tumor targets, PGE2 was added exogenously to the culture.

**Figure 2:**
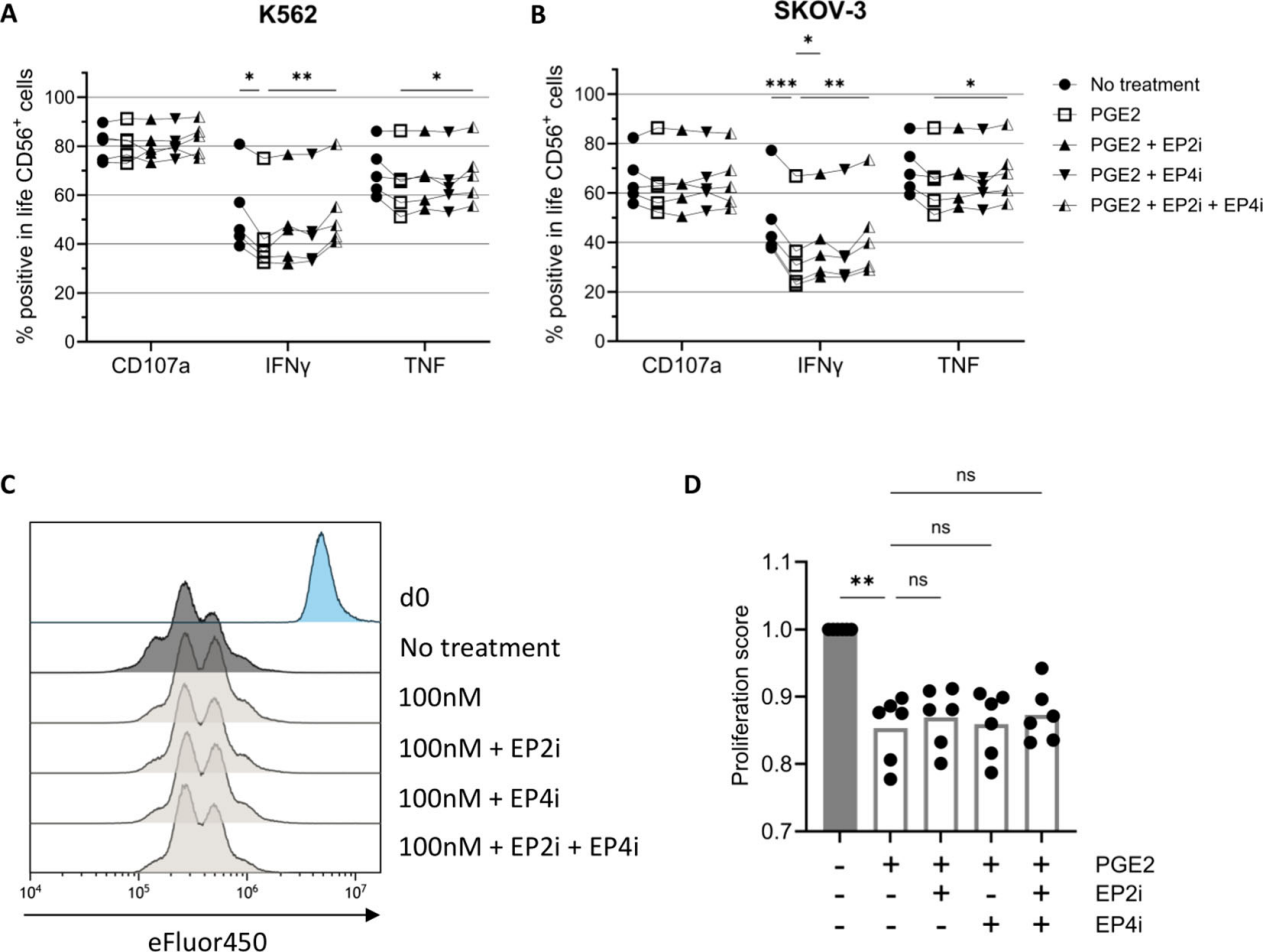
PGE2 suppresses HSPC-NK cell functionality in an EP2 and EP4 receptor-dependent manner. **(A, B)** HSPC-NK cells (N = 5) were stimulated (or not) for 24h with either PGE2 alone, PGE2 in combination with EP2 antagonist, in combination with EP4 antagonist, or in combination with both inhibitors before co-culturing them with **(A)** K562 or **(B)** SKOV-3 to study surface expression of CD107a and production of IFNγ and TNF. Each line represents one donor. **(C)** Representative flow cytometry histograms showing the different degrees of eFluor450 dye dilution upon HSPC-NK cell divisions stimulated with 20ng/mL IL-15 and treated with (or without) 100nM PGE2 and EP2 and/or EP4 inhibitors. **(D)** Proliferation of HSPC-NK cells (N = 6) cultured for 4 days with either PGE2 alone or in combination with EP2 inhibitor, EP4 inhibitor, or both inhibitors simultaneously, and measured by the fold decrease in the MFI of the proliferation dye eFluor450 compared to plating day 0. Bars represent mean values ns = no significance, *p < 0.05, **p < 0.01, ***p < 0.001; **(A)** and **(B)** Two-way ANOVA with Dunnett correction, **(E)** Kruskal-Wallis test with Dunnett correction.

Following our previous studies, we next employed EP2- (AH6809) and EP4- (L-161982) specific antagonists to improve the functionality of HSPC-NK cells (30, 45). First, we studied the functionality of HSPC-NK cells in a 4h co-culture assay with K562 cells and SKOV-3 cells. There was no difference in degranulation capacity between PGE2-treated or naïve HSPC-NK cells (**Figures 2A, B**). Nonetheless, PGE2 reduced IFNγ and TNF production when stimulated with tumor cells. Furthermore, blocking of both EP2 and EP4 resulted in almost complete rescue of cytokine production in PGE2-suppressed HSPC-NK cells. However, EP2 and EP4 blockade could not rescue their proliferative capacity after 4 days in culture (**Figures 2C, D**). DMSO treatment alone did not impact the proliferation of PGE2-treated HSPC-NK cells, ruling out the possibility of DMSO-mediated toxicities impairing proliferation rescue by the inhibitors themselves (data not shown). Together, these results suggest that PGE2 suppression of HSPC-NK cells is mediated by EP2 and EP4, but stable and long-term PGE2 blockade cannot be attained with EP2/EP4 competitive inhibitors.

### 3.3 Knockout of EP2 and EP4 stably rescues PGE2-suppressed NK cells’ functionality

In order to generate stable PGE2-resistant HSPC-NK cells with improved therapeutic effect, we next tested a variety of different CRISPR gRNAs targeting EP2 and EP4 in HSPC-NK cells on day 28 of the HSPC-NK cell culture. Based on *in silico* prediction of specificity and efficacy, four different gRNAs targeting the first exon of *PTGER2* and eight different gRNAs targeting the second exon of *PTGER4* were studied (**Supplementary Figure 3A**; **Supplementary Table 1**). DNA sequencing and in silico analysis of day 35 differentiated HSPC-NK cells confirmed successful knockout of *PTGER2* by gRNA2 (**Supplementary Figure 3B**) and *PTGER4* by all gRNAs except gRNA2 (**Supplementary Figure 3C**). Importantly, the in silico SynthegoICE analysis of the percentage of indels correlated with the percentage of mutated gene (**Supplementary Figures 3D–F**), as well as with the percentage of mutated *PTGER2* and *PTGER4* mRNA (**Supplementary Figures 3G–I**), as evaluated with getPCR technique (42). Based on the empirical efficacy and in silico prediction of specificity, we selected gRNA2 targeting *PTGER2* and gRNA4 targeting *PTGER4* for further experiments.

**Figure 3:**
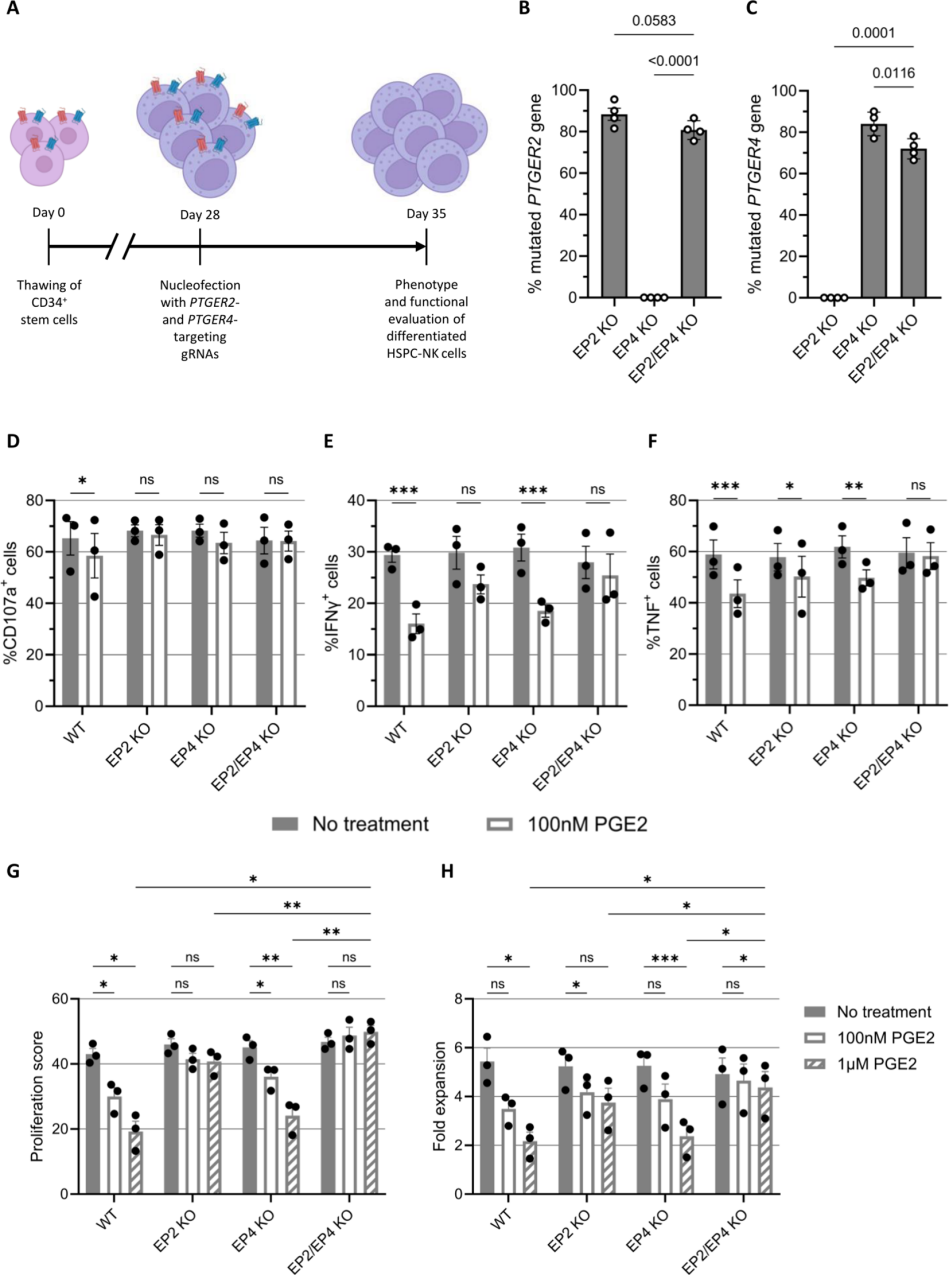
EP2/EP4 KO renders HSPC-NK cells less sensitive to PGE2 inhibition. **(A–C)** HSPC-NK cells (N = 4) on day 28 of the expansion-differentiation protocol were nucleofected with RNP complexes targeting wither *PTGER2* gene (EP2 KO), *PTGER4* gene (EP4 KO) or both (EP2 KO + EP4 KO). Differentiated HSPC-NK cells were assessed for the percentage of mutations in the **(B)** *PTGER2* gene or **(C)** *PTGER4* gene evaluated through SynthegoICE analysis. Bars and error bars represent mean ± SEM, respectively. Numbers represent p-values **(D–F)** Percentage of **(D)** CD107a^+^, **(E)** IFNγ^+^ and **(F)** TNF^+^ HSPC-NK cells (N = 3) knockout (or not) for either or both EP2 and EP4 receptors after 24h treatment with either 100nM PGE2 or not. Bars and error bars represent mean ± SEM, respectively. **(G)** Proliferation and **(H)** expansion of WT, EP2/EP4 single or double KO HSPC-NK cells (N = 3) when subjected to either 100nM or 1μM PGE2 for 6 days, as measured by fold decrease in the MFI of the proliferation dye eFluor450 **(G)** or absolute flow cytometry numbers of 7AAD^–^CD56^+^ HSPC-NK cells **(H)** compared to plating day 0. Bars and error bars represent mean + SEM, respectively. ns = no significance, *p < 0.05, **p < 0.01, ***p < 0.001; **(A, B)** One-way ANOVA with Dunnett correction, **(C–E)** Two-way ANOVA with Sidak correction, **(F, G)** Two-way ANOVA with Tukey correction.

Next, we tested the multiplex knockout of both EP2 and EP4 (EP2/EP4 KO) in our differentiating HSPC-NK cells (**Figure 3A**). We observed an average of 81 ± 5% of knockout of *PTGER2* (**Figure 3B**) and 72 ± 5% of knockout of *PTGER4* (**Figure 3C**) in the double knockout HSPC-NK cell cultures. Notably, neither single nor dual EP2/EP4 KO resulted in significant differences in the NK cell phenotype compared to WT HSPC-NK cells (**Supplementary Figure 4**). Functional assessment of the WT and KO HSPC-NK cells showed that while PGE2-treatment reduced IFNγ and TNF production in WT HSPC-NK, functionality of EP2/EP4 KO HSPC-NK cells was not significantly affected by PGE2 (**Figures 3D–F**). The EP2 or EP4 single KO HSPC-NK cells showed only partial functional rescue. Likewise, single KO of EP2 or EP4 resulted in an improvement in the IL-15-induced proliferative capacity of PGE2-treated HSPC-NK cells compared to WT non-edited cells (**Figures 3G, H**). However, only double EP2/EP4 KO HSPC-NK cells showed full rescue in proliferative capacity as compared to samples not treated with PGE2. We validated that the selected gRNAs could also be used to improve the functionality of alternative NK cell sources, as EP2- and EP4-expressing KHYG-1 cells (**Supplementary Figure 5A**) were effectively knockout for both receptors up to 97% (**Supplementary Figures 5B, C**), resulting in improved antitumor functionality and proliferative capacity (**Supplementary Figures 5D, E**). Collectively, these results demonstrate that both antitumor functionality and proliferation of different NK cell products can be rescued by stable abrogation of the EP2 and EP4 receptor expression through CRISPR KO engineering.

### 3.4 EP2 and EP4 knockout synergizes with tIL15 transduction to improve HSPC-NK cells’ survival in PGE2-enriched, cytokine-deprived environments

NK cells show improved persistence and functionality when stimulated with transpresenting IL-15 (tIL15) mechanism (38, 39). Here, we co-engineered HSPC-NK cells with tIL15 through retroviral transduction to improve the survival, expansion and therapeutic potential of our EP2/EP4 KO HSPC-NK cells. To improve the scalability and translation of the protocol, we attempted to engineer CD34^+^ cells with CRISPR-KO and tIL15 transduction early in the expansion/differentiation protocol and thereafter expand and differentiate the progenitor cells into multi-engineered HSPC-NK cell products. For this, two protocols were considered. First, expanded CD34^+^ cells were CRISPR-modified on day 5 and transduced on day 7 with either tIL15 or GFP (vehicle) retrovirus (**Supplementary Figure 6A**). This resulted in up to 45-fold average expansion from day 7 and successful differentiation to HSPC-NK cells (≥92% CD56^+^), a retroviral vector expression ranging from 8-29%, and stable, high KO percentage of ≥91% for *PTGER2* and ≥83% for *PTGER4* (**Supplementary Figures 6B–G**). In the second protocol, expanded CD34^+^ cells were nucleofected on day 3 and later transduced on the same day (**Supplementary Figure 6H**), resulting in a 261-310-fold average expansion and acquisition of ≥94% CD56^+^ cells. With this protocol, we obtained a transgene expression ranging from 23-54%, and stable and high KO percentage of ≥93% for *PTGER2* and ≥85% *PTGER4* in differentiated HSPC-NK cells (**Supplementary Figures 6I–N**). Notably, both protocols yielded differentiated HSPC-NK cells with minimal differences in phenotype among KO vs. WT or tIL15- vs. GFP-transduced cells (**Supplementary Figures 7A, B**).

**Figure 4:**
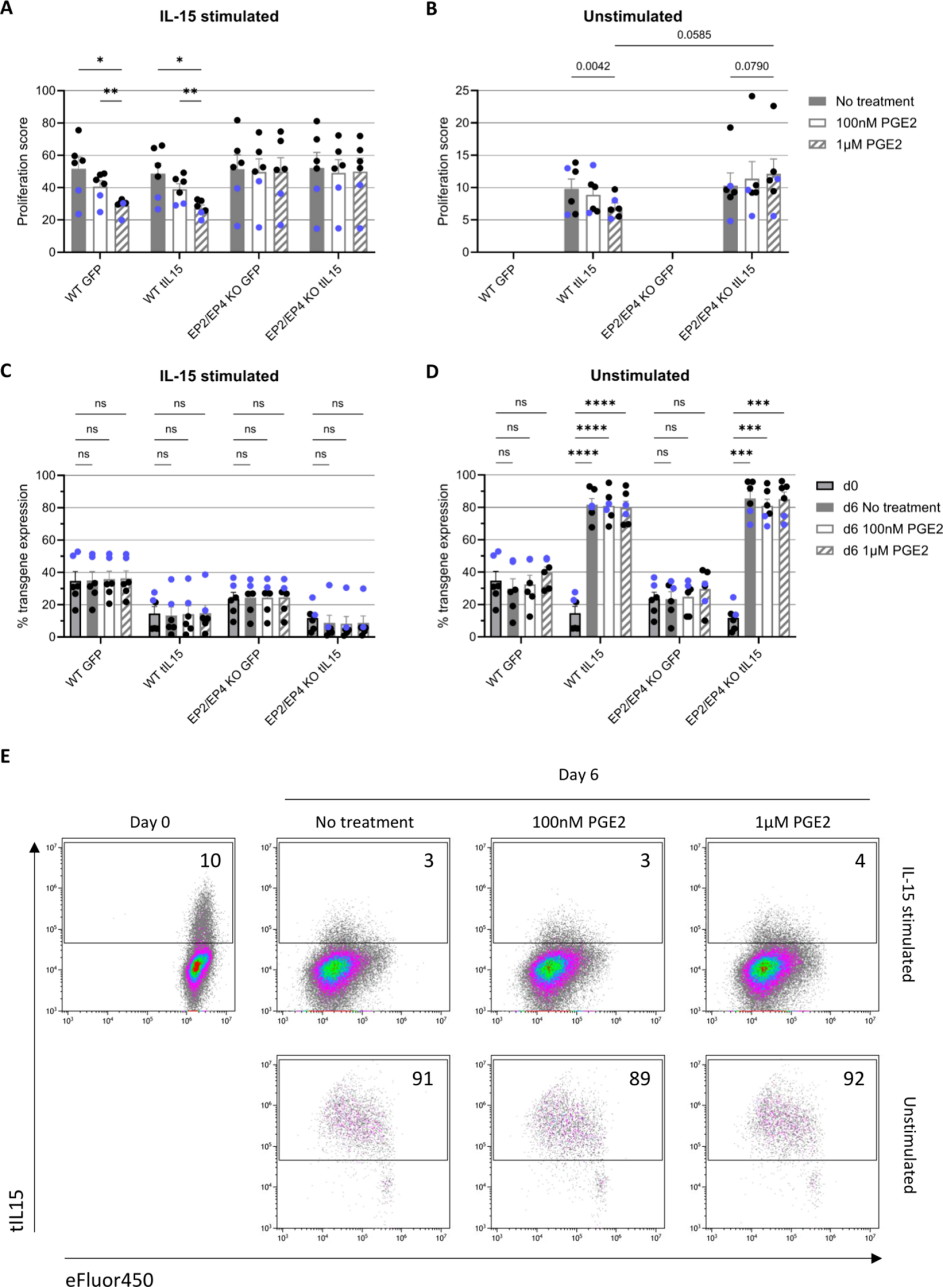
EP2/EP4 KO synergize with tIL15 engineering to improve survival of HSPC-NK cells in PGE2-rich, cytokine-deprived conditions. **(A, B)** Proliferation of HSPC-NK cells (N = 6) after treatment (or not) with 100nM or 1μM PGE2 for 6 days either in the presence of **(A)** 20ng/mL IL-15 or **(B)** in no cytokine supplemented media. Black dots show HSPC-NK cell donors obtained from the expansion-differentiation protocol 1 (N = 4), and blue dots represent HSPC-NK cell donors generated with the expansion-differentiation protocol 2 (N = 2). See also **Supplementary Figure 6**. Numbers in **(B)** indicate p-values. Bars and error bars represent mean + SEM, respectively. **(C, D)** Evolution of the percentage of expression of GFP or surface IL-15Rα, where indicated, in HSPC-NK cells plated for a proliferation assay for six days with or without treatment with 100nM or 1μM of PGE2 in the presence **(C)** or absence **(D)** of exogenous supplementation of 20ng/mL IL-15. Black dots show HSPC-NK cell donors obtained from the expansion-differentiation protocol 1 (N = 4), and blue dots represent HSPC-NK cell donors generated with the expansion-differentiation protocol 2 (N = 2). See also **Supplementary Figure 6**. Bars and error bars represent mean + SEM, respectively. **(E)** Representative flow cytometry dot plot correlating the proliferative capacity (as measured by loss of eFluor450) with tIL15 expression in HSPC-NK cells either stimulated with IL-15 or remained unstimulated for six days, and treated (or not) with the indicated concentrations of PGE2. Numbers in plots represent percentages of tIL15^+^ cells. *p < 0.05, **p < 0.01; **(A–D)** Two-way ANOVA with Dunnett correction. ***p < 0.001, ****p < 0.0001

Because IL-15 signaling is known to enhance NK cell function primarily by promoting expansion and survival, we first performed an *in vitro* proliferation assay to evaluate these effects. As seen before, EP2/EP4 KO improved resistance to PGE2-mediated suppression of proliferation in media supplemented with IL-15 (**Figure 4A**; **Supplementary Figure 8A**). Importantly, only tIL15-transduced HSPC-NK cells survived *in vitro* for up to 6 days without any cytokine supplementation (**Figure 4B**; **Supplementary Figure 8B**). Here, EP2/EP4 KO also improved resilience to PGE2, indicating a synergistic effect of both tIL15 engineering and ablation of PGE2 receptors in enhancing HSPC-NK *in vitro* persistence. In addition, although no significant changes in transgene expression were observed upon IL-15 stimulation after six days, tIL15-transduced cells, but not GFP-transduced counterparts, were positively selected when cultured in cytokine-deprived medium, increasing in frequency from 11-14% before plating to 80-85% average percentage (**Figures 4C, D**). This enrichment was accompanied by a mild upregulation of LAG-3, PD-1 and TIGIT, but not TIM-3 (**Supplementary Figures 8C, D**). Notably, PGE2 treatment did not significantly affect tIL15 enrichment. Moreover, tIL15^bright^ cells proliferated more than tIL15^dim^ or tIL15^–^ counterparts (**Figure 4E**), indicating that the degree of tIL15 expression correlates with better *in vitro* persistence. Overall, co-engineering with EP2/EP4 KO and tIL15 improves survival of HSPC-NK cells when treated with PGE2 without exogenous cytokine supplementation. Given the improved fold expansion and transgene stability of the HSPC-NK cells generated from the second protocol, these cells were selected for further functional evaluation.

### 3.5 HSPC-NK cells’ antitumor functionality in PGE2-enriched, cytokine-deprived environments is improved when co-engineering with EP2 and EP4 knockout and tIL15 transduction

**Figure 5:**
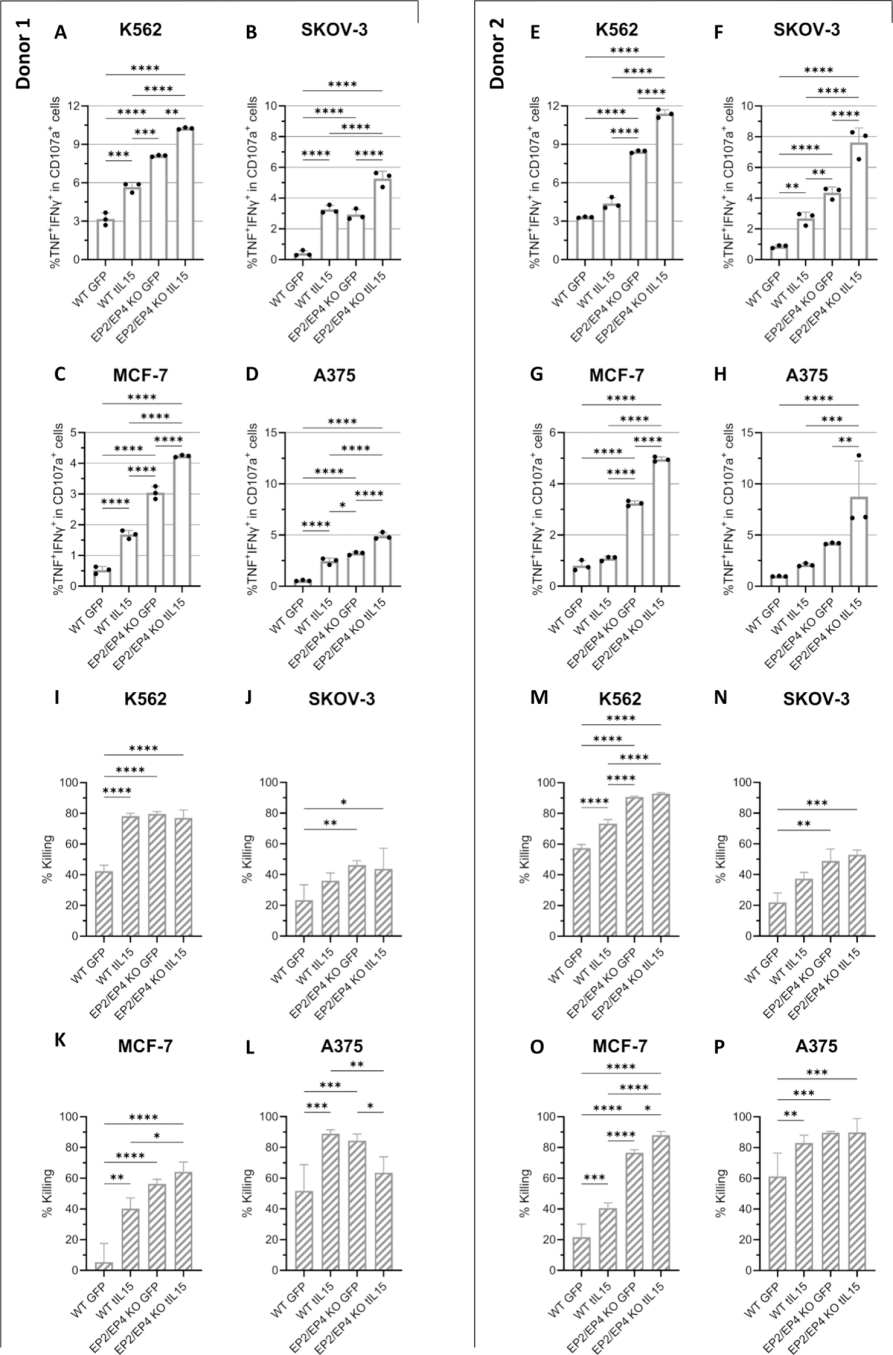
EP2/EP4 KO synergize with tIL15 engineering to render HSPC-NK cells resistant to PGE2 under cytokine-deprived conditions. **(A–H)** Percentage of double IFNγ^+^TNF^+^ within CD107a^+^ HSPC-NK cells co-cultured for 4h with K562, SKOV-3, MCF-7 and A375 of HSPC-NK cell donors 1 **(A–D)** and 2 **(E–H)** after 20h of pre-treatment with 100nM PGE2 and no cytokine supplementation. Data comes from three independent replicates. Bars and error bars represent mean + SD. **(I–P)** Percentage killing of K562, SKOV-3, MCF-7 and A375 cells after 40h co-culture with HSPC-NK cell donors 1 **(I–L)** and 2 **(M–P)** from protocol 2 in media containing 1μM PGE2 and no cytokine supplementation. Data comes from three independent replicates. Bars and error bars represent mean + SD. *p < 0.05, **p < 0.01, ***p < 0.001, ****p < 0.0001; **(A–P)** Two-way ANOVA with Tukey correction.

Because we aimed at generating functionally superior HSPC-NK cells against a wide range of tumors showing an association between PGE2 production and low prognosis or survival, we next sought to further include other tumor models for our functional assays. For this reason, MCF-7, a breast cancer model (46), and A375, a melanoma model (30), with reported PGE2 secretion capabilities were included. Here, we first evaluated the anti-tumor functionality by pre-treating HSPC-NK cells with PGE2 for 20h but without IL-15 supplementation, to evaluate the impact of tIL15 engineering in PGE2 sensitivity. Superior resistance to PGE2 of EP2/EP4 KO and tIL15 engineering of HSPC-NK cells was observed in a potency assay against K562, SKOV-3, MCF-7 and A375 target cells (**Supplementary Figures 9A–H**). More importantly, tIL15 engineering alone or EP2/EP4 KO alone generally resulted in superior polyfunctionality, measured by CD107a^+^IFNγ^+^TNF^+^ expression, compared to WT GFP cells treated with PGE2 (**Figures 5A–H**). In addition, the combination of both EP2/EP4 KO with tIL15 engineering excelled HSPC-NK cells’ polyfunctionality in presence of PGE2 against all tumor models compared to single tIL15 or EP2/EP4 KO engineered cells and WT GFP HSPC-NK (**Figures 5A–H**). This indicates a synergistic effect of co-engineering in driving more potent HSPC-NK cell activation upon target cell recognition. Notably, PGE2-treated EP2/EP4 KO tIL15 HSPC-NK cells showed complete functional rescue when compared to non-treated WT GFP HSPC-NK cells (**Supplementary Figures 9A–H**).

Similar functional effects were observed in a 40h-long killing assay. Both HSPC-NK cell donors engineered with tIL15 killed target cells as efficiently as, and up to 40% more than, the WT GFP-engineered counterparts (**Supplementary Figures 10A–H**). Similarly, all targets were killed more efficiently by WT tIL15 HSPC-NK cells compared to WT GFP HSPC-NK cells when treated with PGE2 (**Figures 5I–P**), indicating that tIL15 stimulation improves HSPC-NK cells’ antitumor functionality in the presence of this immunosuppressive lipid mediator. Importantly, EP2/EP4 KO GFP HSPC-NK cells showed enhanced killing capacity against all target cells under PGE2 treatment compared to WT GFP counterparts (**Figures 5I–P**). In fact, non-treated EP2/EP4 KO GFP HSPC-NK cells showed comparable cytotoxicity to PGE2-treated counterparts (**Supplementary Figures 10A–H**), substantiating that EP2/EP4 KO improves resistance to PGE2-supressed killing capacity. Likewise, EP2/EP4 KO-tIL15-HSPC-NK cells showed an overall improved killing capacity compared to WT GFP and WT tIL15 under PGE2 conditions, but not EP2/EP4 KO GFP (**Figures 5I–P**), indicating that the observed improved cytotoxic capacity at a 0.5:1 effector-to-target ratio after 40h is mainly mediated by EP2/EP4 KO. Altogether, these results demonstrate that PGE2 suppression can be tackled by ablating EP2 and EP4 expression in NK cells, and co-engineering with tIL15 renders cells with improved antitumor functionality even without exogenous cytokine stimulation.

## 4 Discussion

Multiple studies have associated intratumoral NK cell numbers with improved survival in different cancer types (3). In this context and given their natural anti-tumor activity and safety profile, NK cell-based therapies have gained interest for adoptive cell transfer of cancer (47). For this reason, we have developed a protocol for the generation of billions of allogeneic NK cells from hematopoietic stem and progenitor cells (HSPC-NK cells) obtained from umbilical cord blood of neonates (5). However, full efficacy of NK cell therapies is hampered by the suppressive tumor microenvironment, which is especially strong in solid tumors (10). From all immunosuppressive factors, there is compelling evidence associating either increased PGE2 production or upregulation of PGE2-producing enzymes with overall worse prognosis and reduced survival in multiple cancers (12–29, 48). In addition, a recent study used PGE2 alone to mimic tumor-mediated suppression of NK cell function (49), supporting its key role in cancer-associated immunosuppression (50). Therefore, we hypothesized that tackling PGE2-mediated suppression in our HSPC-NK cell product could be an attractive strategy to significantly boost treatment outcomes in cancer patients.

Whilst many studies conducted with both murine (44, 51, 52) and human NK cells (33) have determined EP4 to be the dominant mediator of PGE2 inhibition, others showed that both EP2 and EP4 similarly drive PGE2-mediated suppression in mouse (31) and human NK cells (35, 53). Given these inconsistencies and considering that our HSPC-NK cell products phenotypically differ from naturally-occurring human NK cells (5), we first sought to study the expression profile of the PGE2-interacting EP receptors in our HSPC-NK cell products. As shown previously in human NK cells, we observed no clear expression of EP1 and EP3 at the transcript level with qPCR (35). However, in contrast to other studies using alternative NK cell models, our HSPC-NK cells show higher expression of EP2 than EP4. Despite this, the expression of these PGE2 receptors in HSPC-NK cells warranted further evaluation of their PGE2 sensitivity and strategies to revert such suppression.

Data derived from the functional assays with EP2 and EP4 antagonists, as well as from the EP2 and EP4 single CRISPR KO studies, revealed a dominant role of EP2 in driving PGE2-mediated suppression in HSPC-NK cells. However, the potential role of EP4 in inducing HSPC-NK cell dysfunction upon infusion should not be underestimated. Others have substantiated that EP4 is predominantly expressed in tumor-infiltrating human NK cells (53), and can be upregulated in human peripheral blood NK cells when subjected to cancer cells’ conditioned media *in vitro* (35), thus suggesting that EP4 expression might be upregulated upon infusion of our HSPC-NK cells. In addition, PGE2 affinity for EP4 (Kd = 0.59-1.12nM) (54–56) is higher than that of EP2 (Kd = 13nM) (54, 57). As a minimal upregulation of EP4 expression might have a significant effect in driving PGE2-mediated suppression of our HSPC-NK cells, we decided to block the functionality of both EP2 and EP4. Considering the lack of significant rescue of the proliferative capacity observed with EP2 and EP4 inhibitors, and given the low half-life of up to 12h reported in alternative EP2 and EP4 antagonists (32, 58), we aimed to use CRISPR KO technology to more stably ablate the expression of both receptors in our HSPC-NK cells. Importantly, KO of EP2 and/or EP4 has been previously tested in equine mesenchymal stem cells (59), mice tumors (60), human neuroblastoma cells *in vitro* (61), and in CAR-T cells for pancreatic cancer (62), but, to the best or our knowledge, has never been studied to improve NK cell functionality for adoptive transfer. Here, we report full rescue of the *in vitro* functionality of our HSPC-NK cells upon KO of both PGE2 receptors, which could potentially improve their therapeutic effect for cancer treatment.

Notably, another major hurdle limiting the clinical efficacy of allogeneic NK cells therapies is their low persistence upon infusion (11). Such setback could be tackled with IL-15 stimulation, as this cytokine is well known for improving survival and expansion in NK cells (63). Therefore, we next sought to combine the EP2/EP4 KO with viral engineering with IL-15 and IL-15Rα (tIL15), as we previously demonstrated that transient expression of the IL-15 transpresentation mechanism enhances survival and proliferation of our HSPC-NK cells to a greater extent than IL-15 cytokine alone (38). Here, we showed that only tIL15-engineered cells survived the absence of cytokine support up to six days. More importantly, the tIL15^+/bright^ fraction of engineered HSPC-NK cells was positively selected and ultimately constituted the predominant population surviving under cytokine-deprived conditions, an effect also reported in other tIL15-engineered NK cell products (64). This marked enrichment suggests that stable tIL-15 expression achieved through genetic engineering may better support HSPC-NK cell survival and persistence compared with transient mRNA-based engineering methods (38). In addition, only 5-10% of the EP2/EP4 KO tIL15-HSPC-NK plated cells were detected after 6 days, which corresponds to the initial 10% fraction of tIL15^+^ cells. Therefore, these data collectively suggest that the tIL-15 transduced cells do not expand uncontrollably, but rather persist longer compared to the GFP counterparts. Notably, although we did not study the persistence of our engineered HSPC-NK cells *in vivo*, others have previously substantiated that superior *in vitro* survival and/or expansion in IL-15-engineered NK cells directly correlate with superior persistence in both tumor-bearing and tumor-free NSG mouse models (65–67). Additionally, further engineering with EP2/EP4 KO improved their survival, potency and cytotoxic capacity upon PGE2 treatment. Given that NK cell stimulating cytokines are generally acknowledged to be present at a low-to-moderate degree in the tumor microenvironment, others have already considered tIL15 engineering to be essential to boost NK cell persistence and ultimately improve treatment outcomes (68). Therefore, we here proved that tIL15 engineering can be used to improve the persistence and therapeutic potential of our HSPC-NK cells.

In concomitance with previous studies showing upregulation of exhaustion markers on NK cells after chronic IL-15 exposure (69, 70), we also observed a moderate upregulation of exhaustion markers in EP2/EP4 KO tIL15-HSPC-NK cells after six days in culture without cytokine stimulation. In addition, others previously showed that continuous IL-15 stimulation might modulate NK cell metabolic fitness (71, 72). Whether such mild upregulation of exhaustion markers and the potential switch of metabolic profile leads to functional impairment, and how co-engineering of IL-15 with IL-15Rα influences these effects is yet unknown. Nevertheless, we here demonstrate that tIL15 engineering is essential to ensure HSPC-NK cells persist under cytokine deprived environments. In addition, various studies have substantiated that the combination of IL-15 with IL-15Rα reduces the toxicity effects reported in IL-15-engineered NK (73) and T cells (74) in preclinical mouse models. Given the controlled persistence profile of our HSPC-NK cells and the reduced toxicity profiles observed when IL-15 is co-engineered with its IL-15Rα, we anticipate that no safety suicide switches are needed as opposed to other soluble IL-15-engineered NK cell products (75, 76). Nonetheless, a final safety evaluation of our EP2/EP4 KO tIL15-HSPC-NK cells in preclinical mouse models is warranted before studying the dose-limiting toxicities in a phase I clinical trial.

Additionally, to ensure regulatory approval of our multi-engineered HSPC-NK cells key analysis should be conducted. Although the gRNAs employed were selected with a low in silico off-target profile, lack of genetic abnormalities and mutations as a result of the multiplex CRISPR-Cas9 knockout procedure should be validated. This includes NGS for indel profiling, karyotyping for the detection of aneuploidy or large deletions/insertions, or CAST-Seq for chromosomal rearrangements or translocations. These and alternative methods have been thoroughly described by Tao J. et al. (77). Alternative CRISPR strategies could be contemplated to reduce off-target risks and improve editing precision, including prime- or base-editing (77). Additionally, to reduce the risks of insertional mutagenesis leading to the activation of oncogenes after viral transduction, a tight control of the vector copy number is critical, which can be evaluated with quantitative PCR (78). Implementation of these analyses is key for the successful translation of the engineered product into the clinic.

Besides the safety concerns, the production complexities of the proposed product should also be carefully considered. We have previously improved manufacturing scalability and clinical implementation of our HSPC-NK cells by demonstrating the therapeutic applicability of cryopreserved products produced in G-rex bioreactors (79). In addition, we are currently validating the feasibility of pooling multiple stem cell donors and optimizing the initial stem cell densities to further increase the fold expansion achieved with our HSPC-NK cell production platform. Furthermore, we preclinically demonstrated the feasibility of performing dual EP2/EP4 KO and tIL15 engineering early in the expansion and differentiation protocol for the generation of HSPC-NK cells, which could simplify and facilitate the clinical translation of this next-generation HSPC-NK cell product. Nonetheless, sequential gene editing steps increase the manufacturing complexity. Notably, lentiviruses and retroviruses have been previously used for the *ex vivo* engineering of NK cells in the clinic (80–82). Likewise, ex vivo engineering of stem cells with CRISPR technology recently received FDA approval (83). However, a tight optimization of the engineering protocol in a GMP-compliant environment is warranted before our multi-engineered HSPC-NK cell product can be clinically implemented. If successful, our EP2/EP4 KO tIL15-HSPC-NK cells could be a promising universal therapeutic strategy for tumors with upregulated expression of PGE2-producing enzymes.

## Data Availability

The raw data supporting the conclusions of this article will be made available by the authors at the Radboud Data Repository with a 10-year embargo. Access to the data is possible upon request to the correspondence author.
