# Correction: Engineering with EP2/EP4 knockout and IL-15 transpresentation renders stem cell-derived NK cells self-persistent and resistant to PGE2 inhibition

**DOI:** 10.3389/fimmu.2026.1920703

**Published:** 2026-07-28

**Authors:** Marcos Vidal-Manrique, Laura Hooijmaijers, Julia A.J. Teders, Elif Saf, Kjell Klarenbeek, Iris M. Hagemans, Jorge Cuenca-Escalona, Alessandra Cambi, I. Jolanda M. de Vries, Paul K. J. D. de Jonge, Anniek B. van der Waart, Harry Dolstra

**Affiliations:** 1 Department of Laboratory Medicine, Laboratory of Hematology, Radboud University Medical Center, Nijmegen, Netherlands; 2 Department of Medical BioSciences, Radboud University Medical Center, Nijmegen, Netherlands

**Keywords:** adoptive cell transfer, CRISPR, hematopoietic stem cells, IL-15 transpresentation, NK cells, prostaglandin E2

The **Data availability statement** was erroneously given as “The authors acknowledge that the data presented in this study must be deposited and made publicly available in an acceptable repository, prior to publication. Frontiers cannot accept an article that does not adhere to our open data policies.” The correct **Data availability statement** is “The raw data supporting the conclusions of this article will be made available by the authors at the Radboud Data Repository with a 10-year embargo. Access to the data is possible upon request to the correspondence author.”

The **Author contributions** statement was erroneously given as “OT: Conceptualization, Data curation, Formal analysis, Investigation, Methodology, Project administration, Software, Visualization, Writing – original draft, Writing – review & editing. YJ: Conceptualization, Data curation, Formal analysis, Investigation, Methodology, Visualization, Writing – review & editing. HW: Conceptualization, Data curation, Formal analysis, Investigation, Methodology, Writing – review & editing. JR: Data curation, Formal analysis, Investigation, Visualization, Writing – review & editing. MP: Data curation, Formal analysis, Investigation, Visualization, Writing – review & editing. JN: Supervision, Validation, Writing – review & editing. MM: Conceptualization, Data curation, Formal analysis, Funding acquisition, Investigation, Methodology, Project administration, Resources, Supervision, Validation, Visualization, Writing – review & editing. ME: Conceptualization, Funding acquisition, Methodology, Project administration, Resources, Supervision, Validation, Writing – review & editing.” The correct **Author contributions** statement is “MV-M: Conceptualization, Methodology, Investigation, Writing—original draft, Writing—review & editing, LH: Investigation, Writing—original draft, Writing—review & editing, JAJT: Investigation, Writing—original draft, Writing—review & editing, ES: Investigation, Writing—original draft, Writing—review & editing, KK: Investigation, Writing—original draft, Writing—review & editing, IMH: Methodology, Writing—original draft, Writing—review & editing, JC-E: Investigation, Writing—original draft, Writing—review & editing, AC: Writing—original draft, Writing—review & editing, Funding acquisition, IJMV: Writing—original draft, Writing—review & editing, Funding acquisition, PKJDJ: Writing—original draft, Writing—review & editing, Funding acquisition, Supervision, ABW: Writing—original draft, Writing—review & editing, Funding acquisition, Supervision, HD: Conceptualization, Writing—original draft, Writing—review & editing, Funding acquisition, Supervision”.

The original version of this article has been updated.

