# Supplementary material for: Engineering with EP2/EP4 knockout and IL-15 transpresentation renders stem cell-derived NK cells self-persistent and resistant to PGE2 inhibition
Source: Front Immunol. 2026 Jun 26;17:1792303. doi: 10.3389/fimmu.2026.1792303 (PMC13350024; doi:10.3389/fimmu.2026.1792303)
Supplement: Supplementary file 1 [file Presentation1.pptx]

## Slide 1
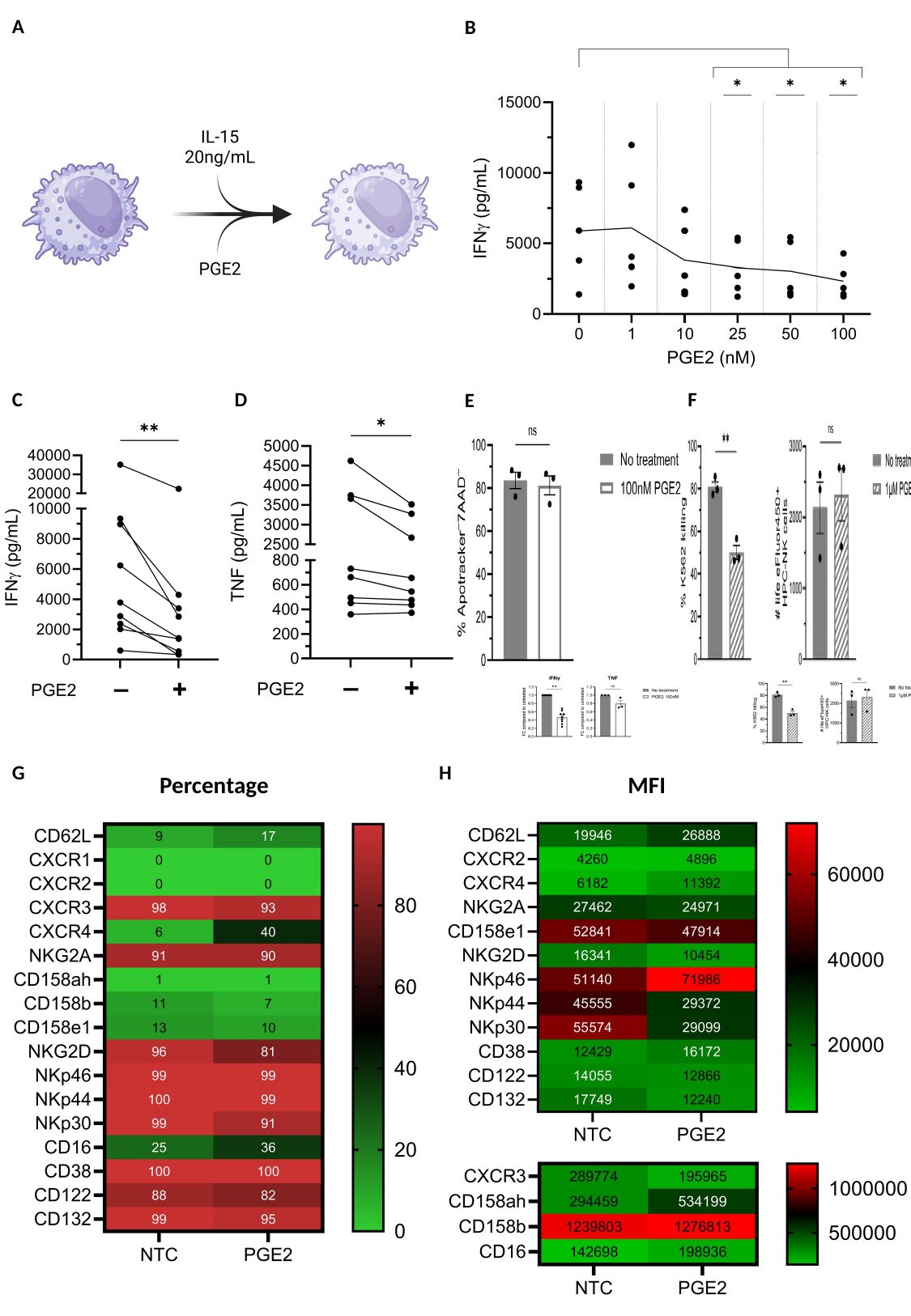

A
B
F
D
C
E
G
H
Percentage
MFI

## Slide 2
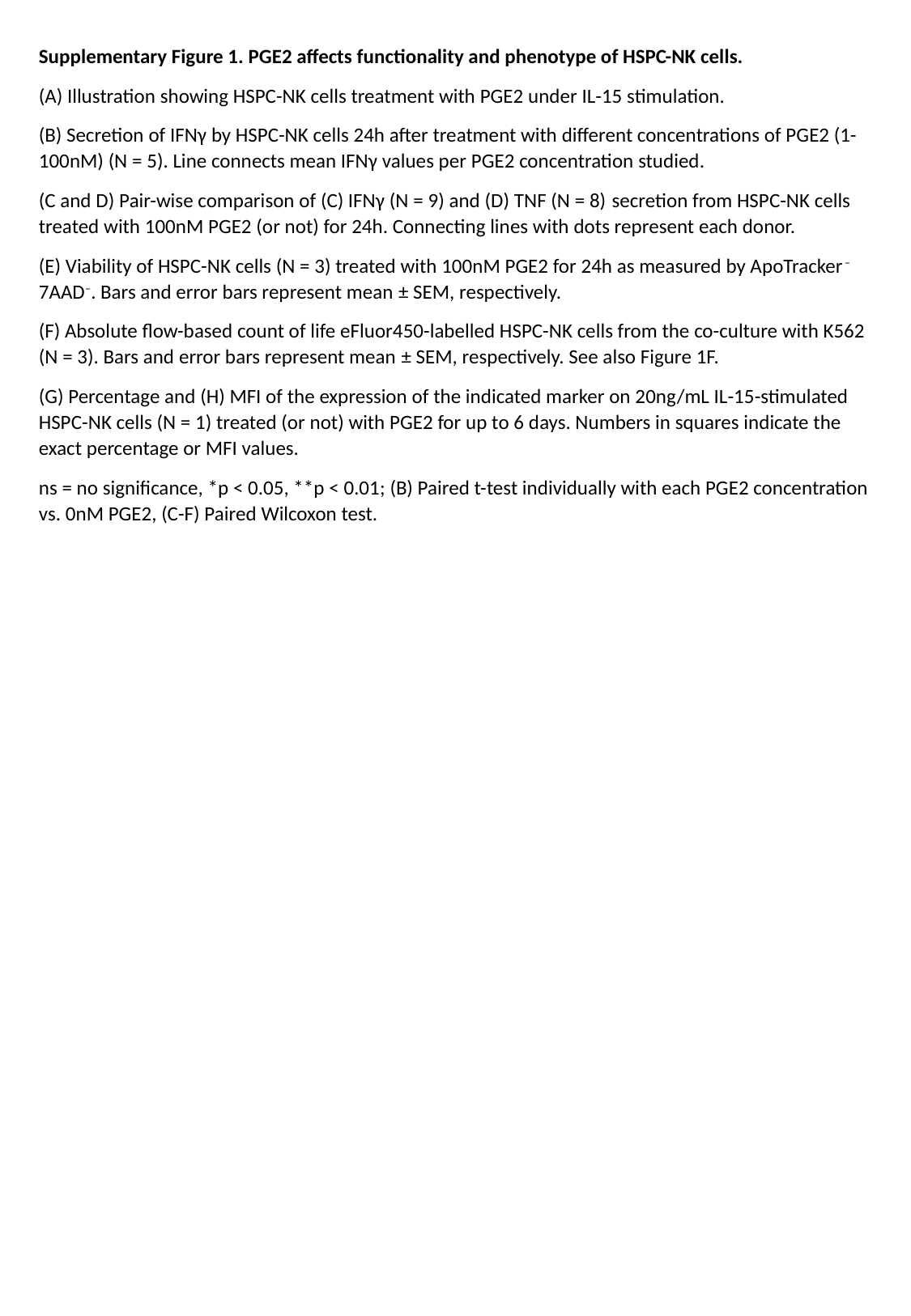

Supplementary Figure 1. PGE2 affects functionality and phenotype of HSPC-NK cells.
(A) Illustration showing HSPC-NK cells treatment with PGE2 under IL-15 stimulation.
(B) Secretion of IFNγ by HSPC-NK cells 24h after treatment with different concentrations of PGE2 (1-100nM) (N = 5). Line connects mean IFNγ values per PGE2 concentration studied.
(C and D) Pair-wise comparison of (C) IFNγ (N = 9) and (D) TNF (N = 8) secretion from HSPC-NK cells treated with 100nM PGE2 (or not) for 24h. Connecting lines with dots represent each donor.
(E) Viability of HSPC-NK cells (N = 3) treated with 100nM PGE2 for 24h as measured by ApoTracker–7AAD–. Bars and error bars represent mean ± SEM, respectively.
(F) Absolute flow-based count of life eFluor450-labelled HSPC-NK cells from the co-culture with K562 (N = 3). Bars and error bars represent mean ± SEM, respectively. See also Figure 1F.
(G) Percentage and (H) MFI of the expression of the indicated marker on 20ng/mL IL-15-stimulated HSPC-NK cells (N = 1) treated (or not) with PGE2 for up to 6 days. Numbers in squares indicate the exact percentage or MFI values.
ns = no significance, *p < 0.05, **p < 0.01; (B) Paired t-test individually with each PGE2 concentration vs. 0nM PGE2, (C-F) Paired Wilcoxon test.

## Slide 3
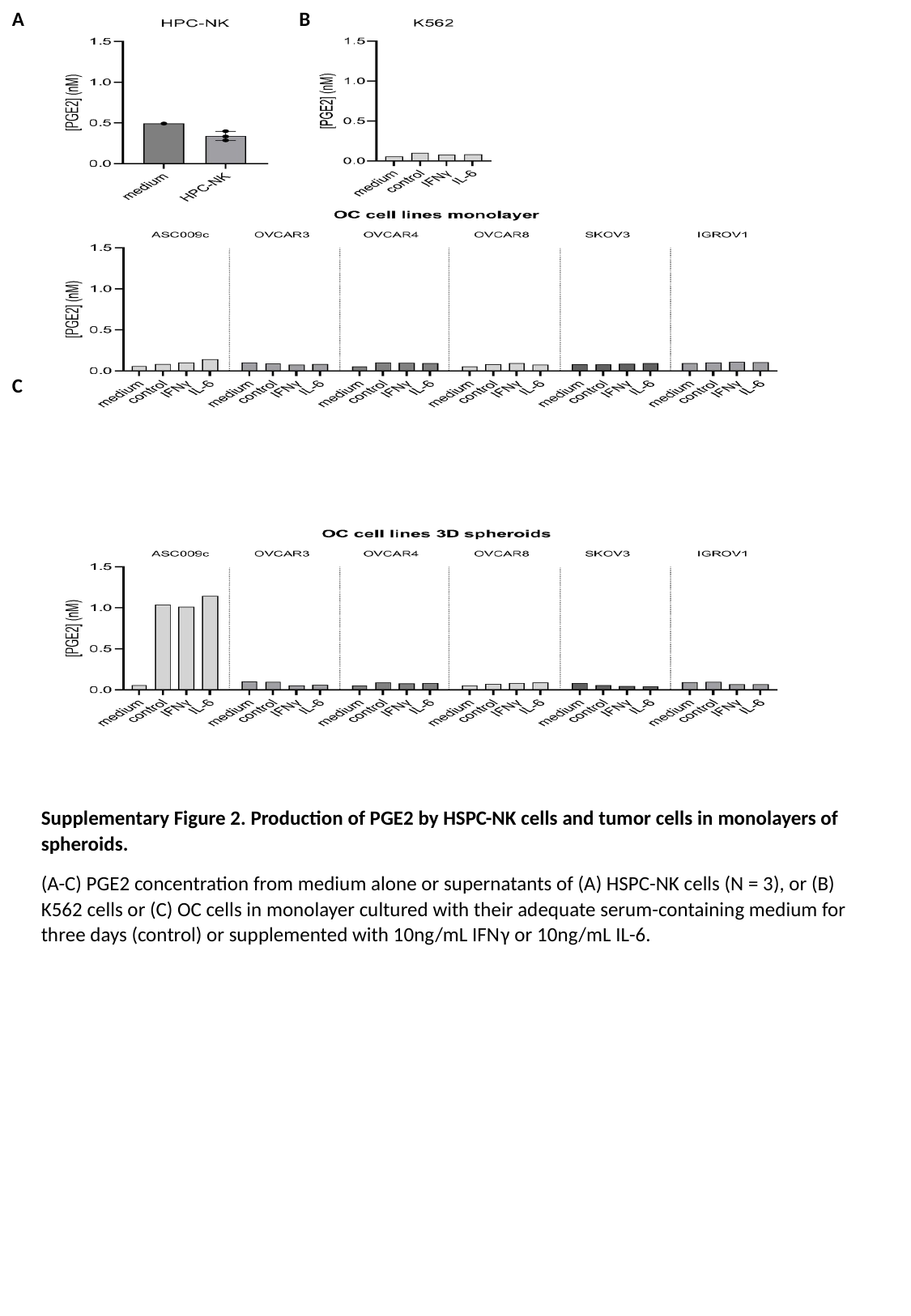

B
A
C
Supplementary Figure 2. Production of PGE2 by HSPC-NK cells and tumor cells in monolayers of spheroids.
(A-C) PGE2 concentration from medium alone or supernatants of (A) HSPC-NK cells (N = 3), or (B) K562 cells or (C) OC cells in monolayer cultured with their adequate serum-containing medium for three days (control) or supplemented with 10ng/mL IFNγ or 10ng/mL IL-6.

## Slide 4
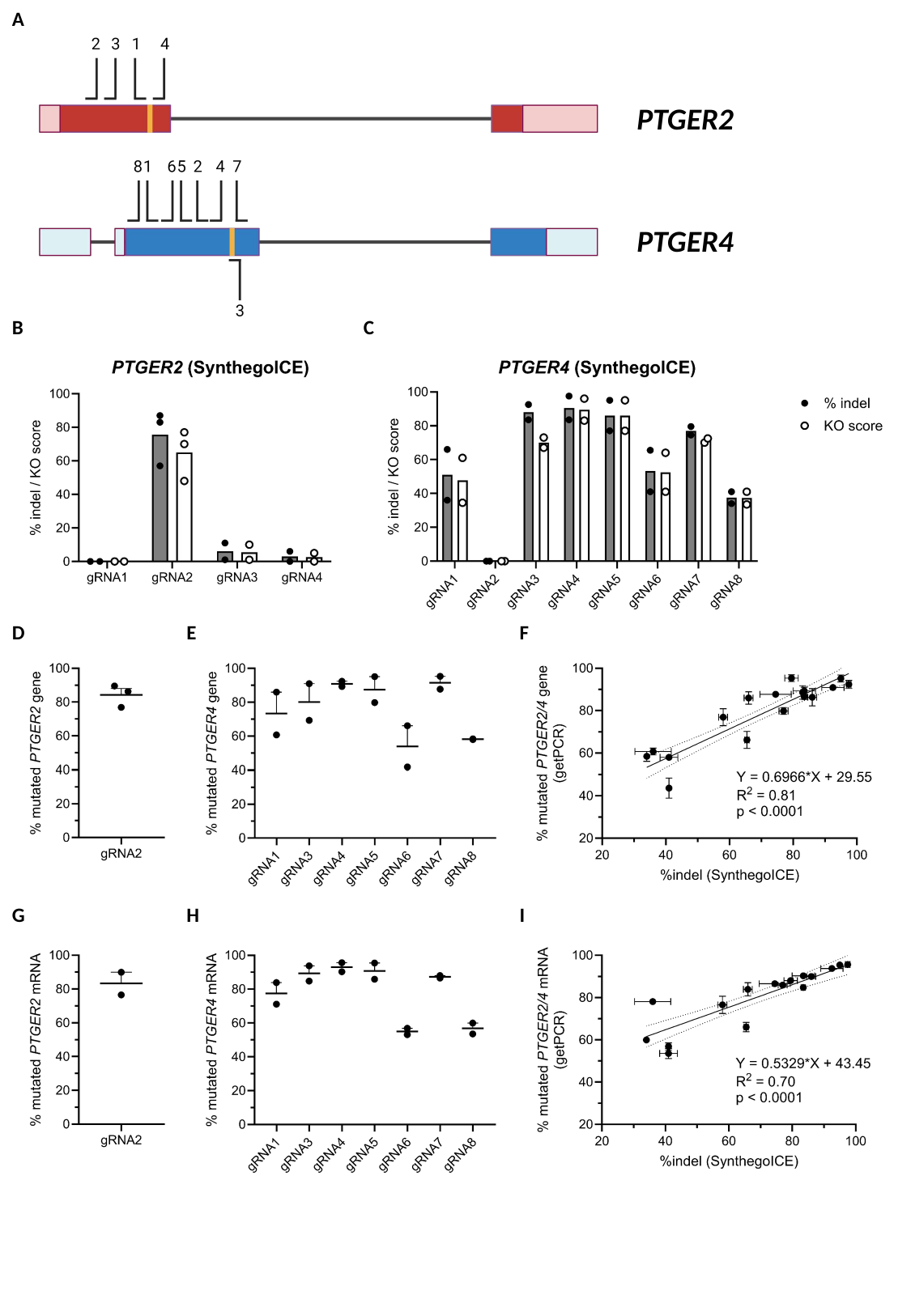

A
PTGER2
PTGER4
B
C
D
E
F
G
H
I

## Slide 5
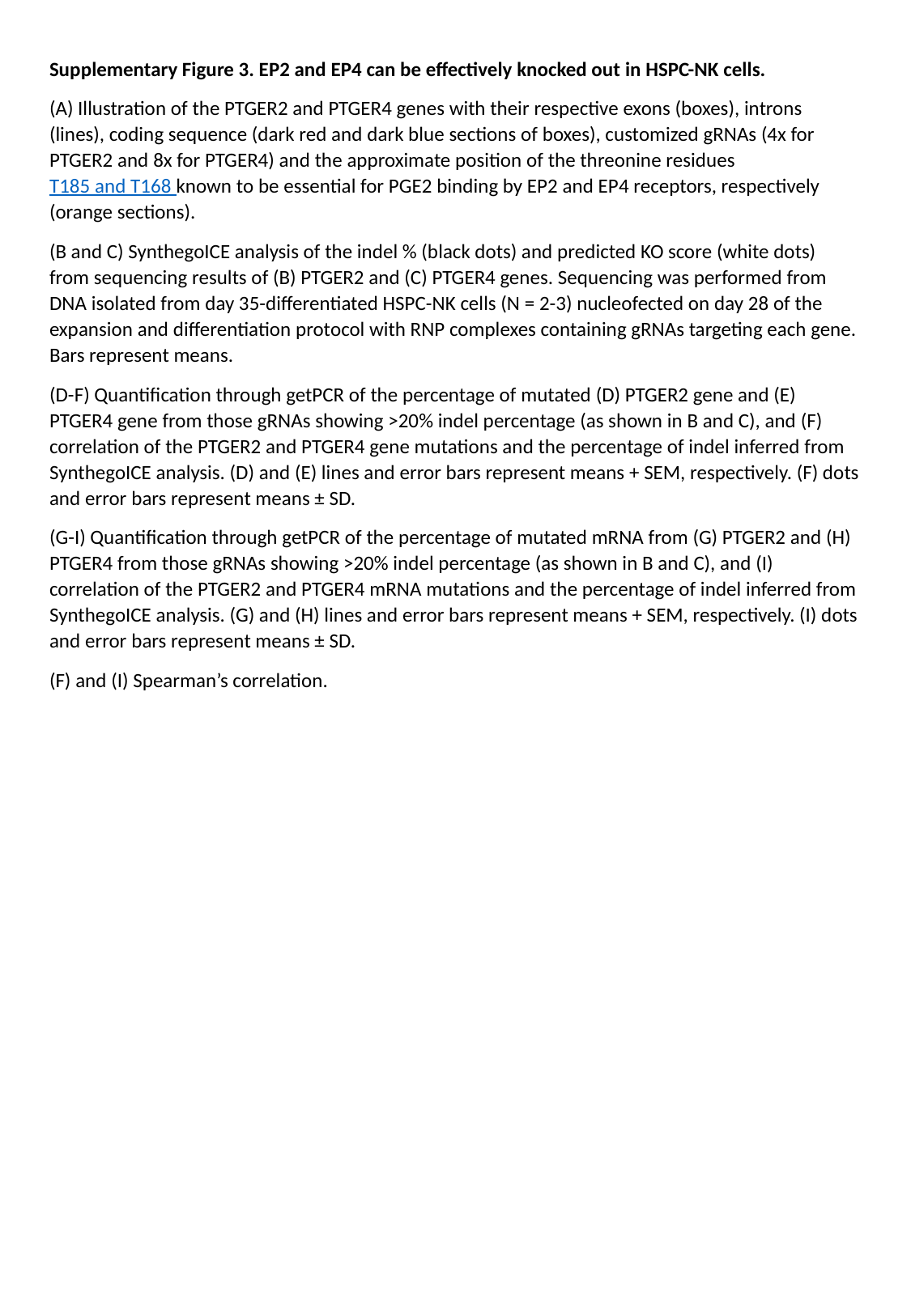

Supplementary Figure 3. EP2 and EP4 can be effectively knocked out in HSPC-NK cells.
(A) Illustration of the PTGER2 and PTGER4 genes with their respective exons (boxes), introns (lines), coding sequence (dark red and dark blue sections of boxes), customized gRNAs (4x for PTGER2 and 8x for PTGER4) and the approximate position of the threonine residues T185 and T168 known to be essential for PGE2 binding by EP2 and EP4 receptors, respectively (orange sections).
(B and C) SynthegoICE analysis of the indel % (black dots) and predicted KO score (white dots) from sequencing results of (B) PTGER2 and (C) PTGER4 genes. Sequencing was performed from DNA isolated from day 35-differentiated HSPC-NK cells (N = 2-3) nucleofected on day 28 of the expansion and differentiation protocol with RNP complexes containing gRNAs targeting each gene. Bars represent means.
(D-F) Quantification through getPCR of the percentage of mutated (D) PTGER2 gene and (E) PTGER4 gene from those gRNAs showing >20% indel percentage (as shown in B and C), and (F) correlation of the PTGER2 and PTGER4 gene mutations and the percentage of indel inferred from SynthegoICE analysis. (D) and (E) lines and error bars represent means + SEM, respectively. (F) dots and error bars represent means ± SD.
(G-I) Quantification through getPCR of the percentage of mutated mRNA from (G) PTGER2 and (H) PTGER4 from those gRNAs showing >20% indel percentage (as shown in B and C), and (I) correlation of the PTGER2 and PTGER4 mRNA mutations and the percentage of indel inferred from SynthegoICE analysis. (G) and (H) lines and error bars represent means + SEM, respectively. (I) dots and error bars represent means ± SD.
(F) and (I) Spearman’s correlation.

## Slide 6
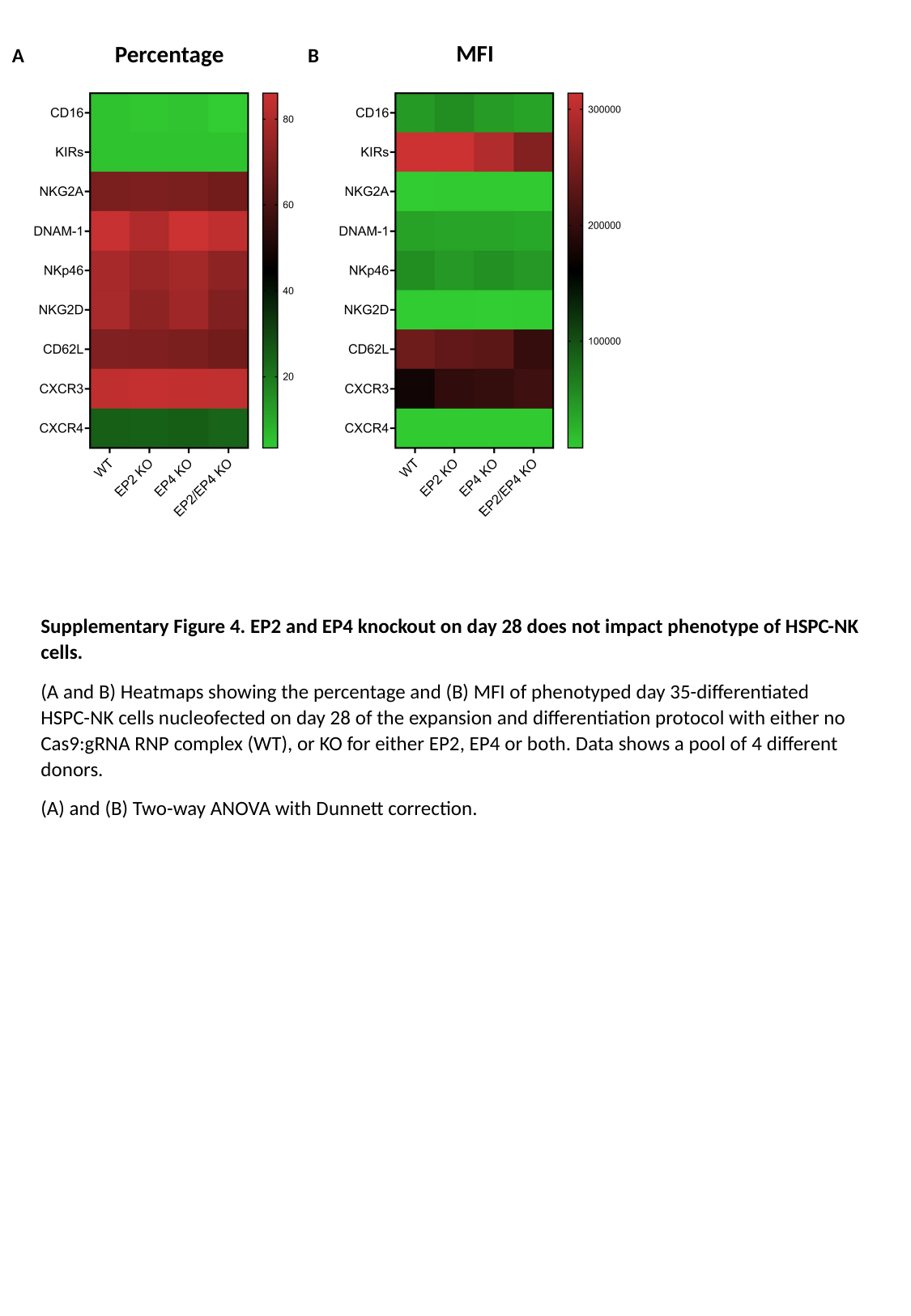

MFI
Percentage
B
A
Supplementary Figure 4. EP2 and EP4 knockout on day 28 does not impact phenotype of HSPC-NK cells.
(A and B) Heatmaps showing the percentage and (B) MFI of phenotyped day 35-differentiated HSPC-NK cells nucleofected on day 28 of the expansion and differentiation protocol with either no Cas9:gRNA RNP complex (WT), or KO for either EP2, EP4 or both. Data shows a pool of 4 different donors.
(A) and (B) Two-way ANOVA with Dunnett correction.

## Slide 7
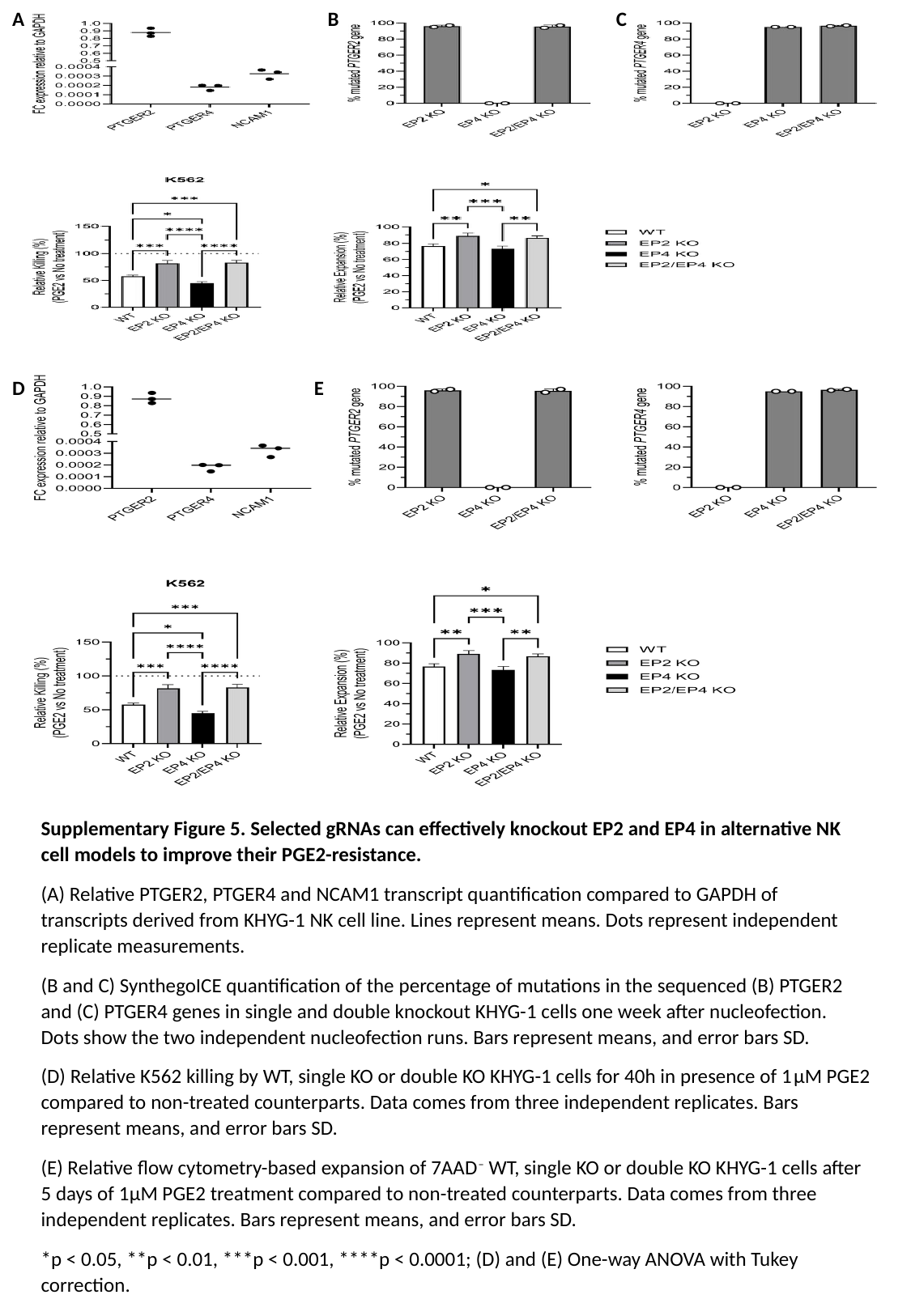

A
B
C
D
E
Supplementary Figure 5. Selected gRNAs can effectively knockout EP2 and EP4 in alternative NK cell models to improve their PGE2-resistance.
(A) Relative PTGER2, PTGER4 and NCAM1 transcript quantification compared to GAPDH of transcripts derived from KHYG-1 NK cell line. Lines represent means. Dots represent independent replicate measurements.
(B and C) SynthegoICE quantification of the percentage of mutations in the sequenced (B) PTGER2 and (C) PTGER4 genes in single and double knockout KHYG-1 cells one week after nucleofection. Dots show the two independent nucleofection runs. Bars represent means, and error bars SD.
(D) Relative K562 killing by WT, single KO or double KO KHYG-1 cells for 40h in presence of 1μM PGE2 compared to non-treated counterparts. Data comes from three independent replicates. Bars represent means, and error bars SD.
(E) Relative flow cytometry-based expansion of 7AAD– WT, single KO or double KO KHYG-1 cells after 5 days of 1μM PGE2 treatment compared to non-treated counterparts. Data comes from three independent replicates. Bars represent means, and error bars SD.
*p < 0.05, **p < 0.01, ***p < 0.001, ****p < 0.0001; (D) and (E) One-way ANOVA with Tukey correction.

## Slide 8
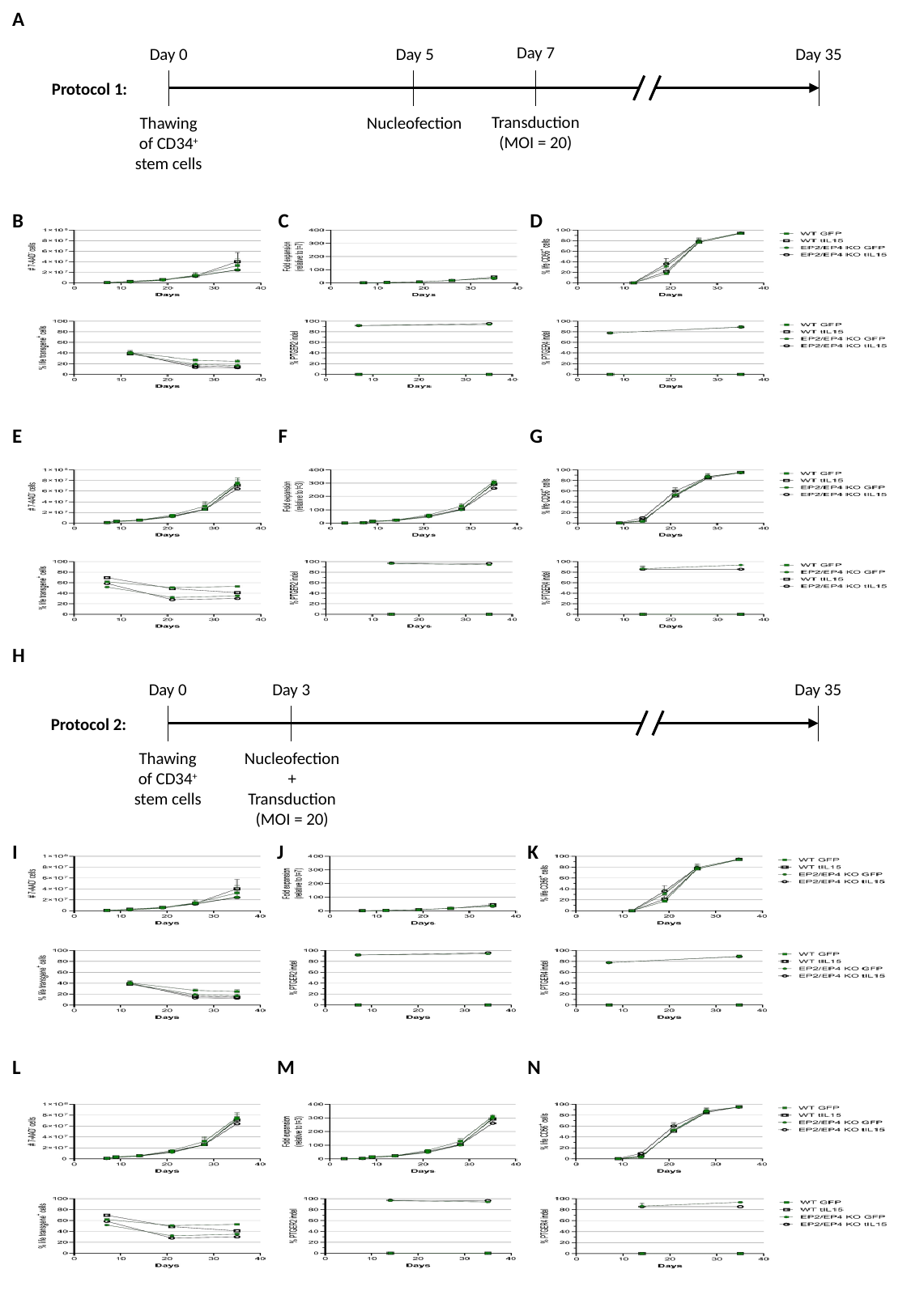

A
Day 7
Day 0
Day 5
Day 35
Protocol 1:
Transduction
(MOI = 20)
Thawing
of CD34+ stem cells
Nucleofection
C
D
B
E
F
G
H
Day 0
Day 3
Day 35
Protocol 2:
Thawing
of CD34+ stem cells
Nucleofection
+
Transduction
(MOI = 20)
J
K
I
L
M
N

## Slide 9
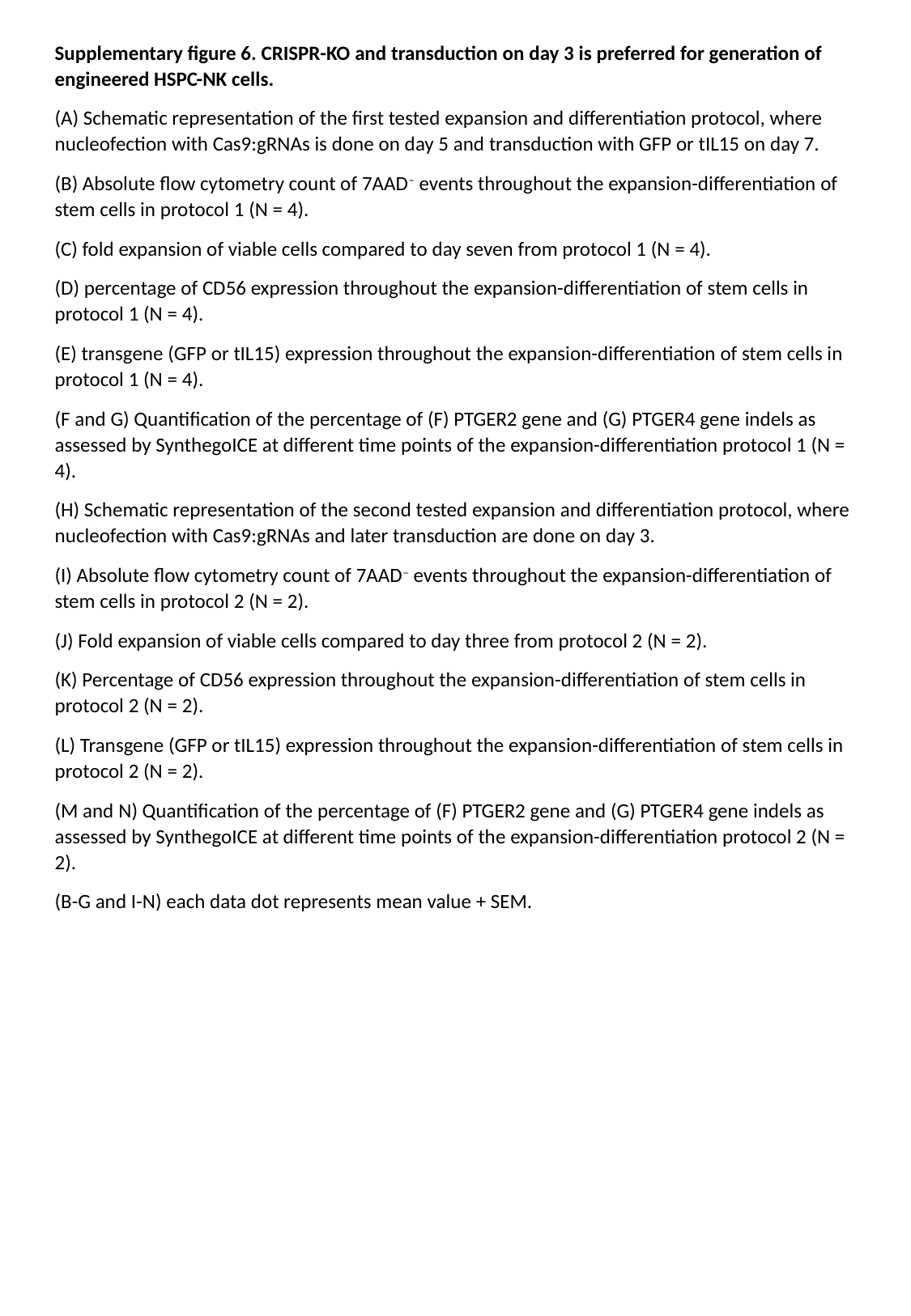

Supplementary figure 6. CRISPR-KO and transduction on day 3 is preferred for generation of engineered HSPC-NK cells.
(A) Schematic representation of the first tested expansion and differentiation protocol, where nucleofection with Cas9:gRNAs is done on day 5 and transduction with GFP or tIL15 on day 7.
(B) Absolute flow cytometry count of 7AAD– events throughout the expansion-differentiation of stem cells in protocol 1 (N = 4).
(C) fold expansion of viable cells compared to day seven from protocol 1 (N = 4).
(D) percentage of CD56 expression throughout the expansion-differentiation of stem cells in protocol 1 (N = 4).
(E) transgene (GFP or tIL15) expression throughout the expansion-differentiation of stem cells in protocol 1 (N = 4).
(F and G) Quantification of the percentage of (F) PTGER2 gene and (G) PTGER4 gene indels as assessed by SynthegoICE at different time points of the expansion-differentiation protocol 1 (N = 4).
(H) Schematic representation of the second tested expansion and differentiation protocol, where nucleofection with Cas9:gRNAs and later transduction are done on day 3.
(I) Absolute flow cytometry count of 7AAD– events throughout the expansion-differentiation of stem cells in protocol 2 (N = 2).
(J) Fold expansion of viable cells compared to day three from protocol 2 (N = 2).
(K) Percentage of CD56 expression throughout the expansion-differentiation of stem cells in protocol 2 (N = 2).
(L) Transgene (GFP or tIL15) expression throughout the expansion-differentiation of stem cells in protocol 2 (N = 2).
(M and N) Quantification of the percentage of (F) PTGER2 gene and (G) PTGER4 gene indels as assessed by SynthegoICE at different time points of the expansion-differentiation protocol 2 (N = 2).
(B-G and I-N) each data dot represents mean value + SEM.

## Slide 10
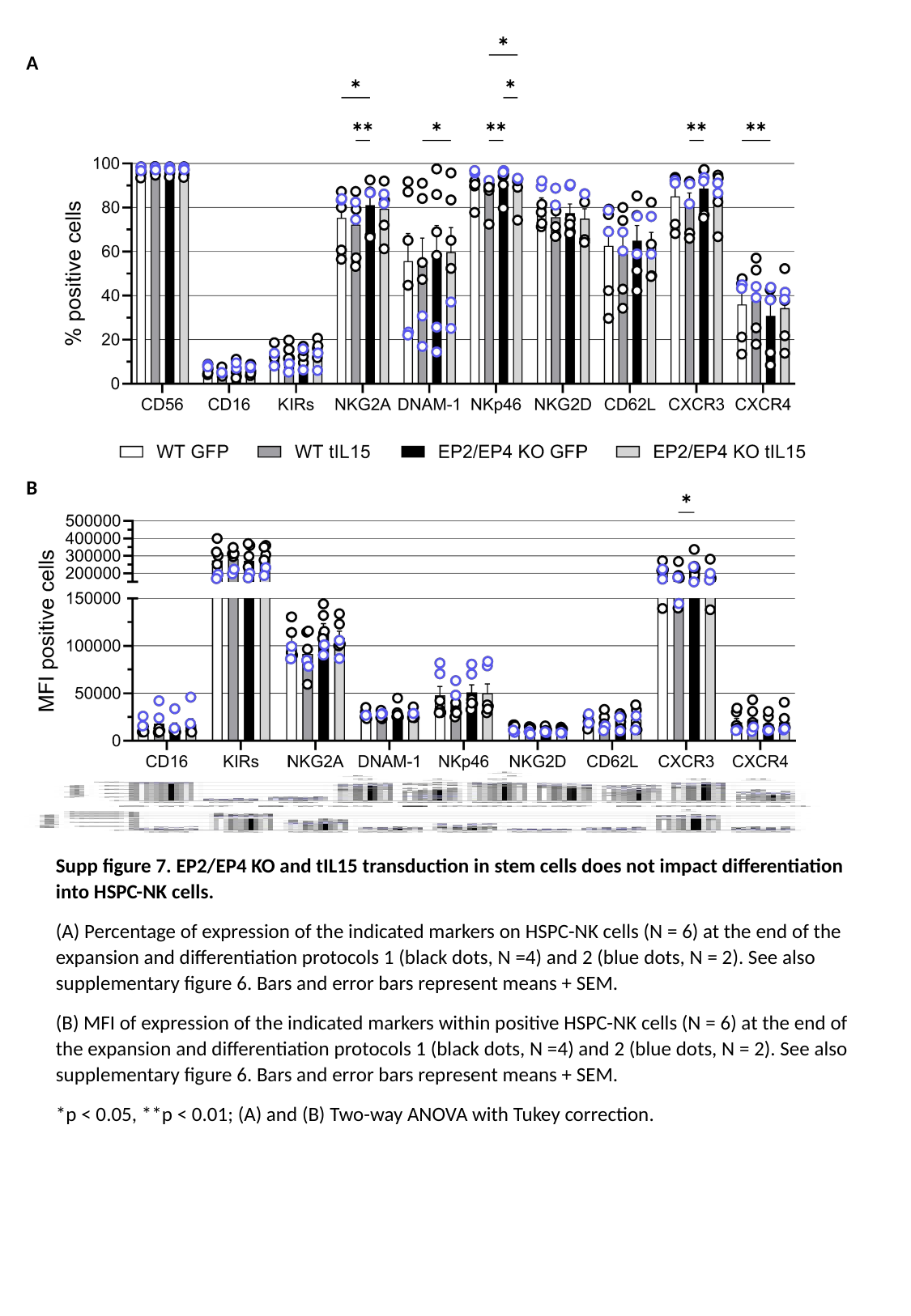

A
B
Supp figure 7. EP2/EP4 KO and tIL15 transduction in stem cells does not impact differentiation into HSPC-NK cells.
(A) Percentage of expression of the indicated markers on HSPC-NK cells (N = 6) at the end of the expansion and differentiation protocols 1 (black dots, N =4) and 2 (blue dots, N = 2). See also supplementary figure 6. Bars and error bars represent means + SEM.
(B) MFI of expression of the indicated markers within positive HSPC-NK cells (N = 6) at the end of the expansion and differentiation protocols 1 (black dots, N =4) and 2 (blue dots, N = 2). See also supplementary figure 6. Bars and error bars represent means + SEM.
*p < 0.05, **p < 0.01; (A) and (B) Two-way ANOVA with Tukey correction.

## Slide 11
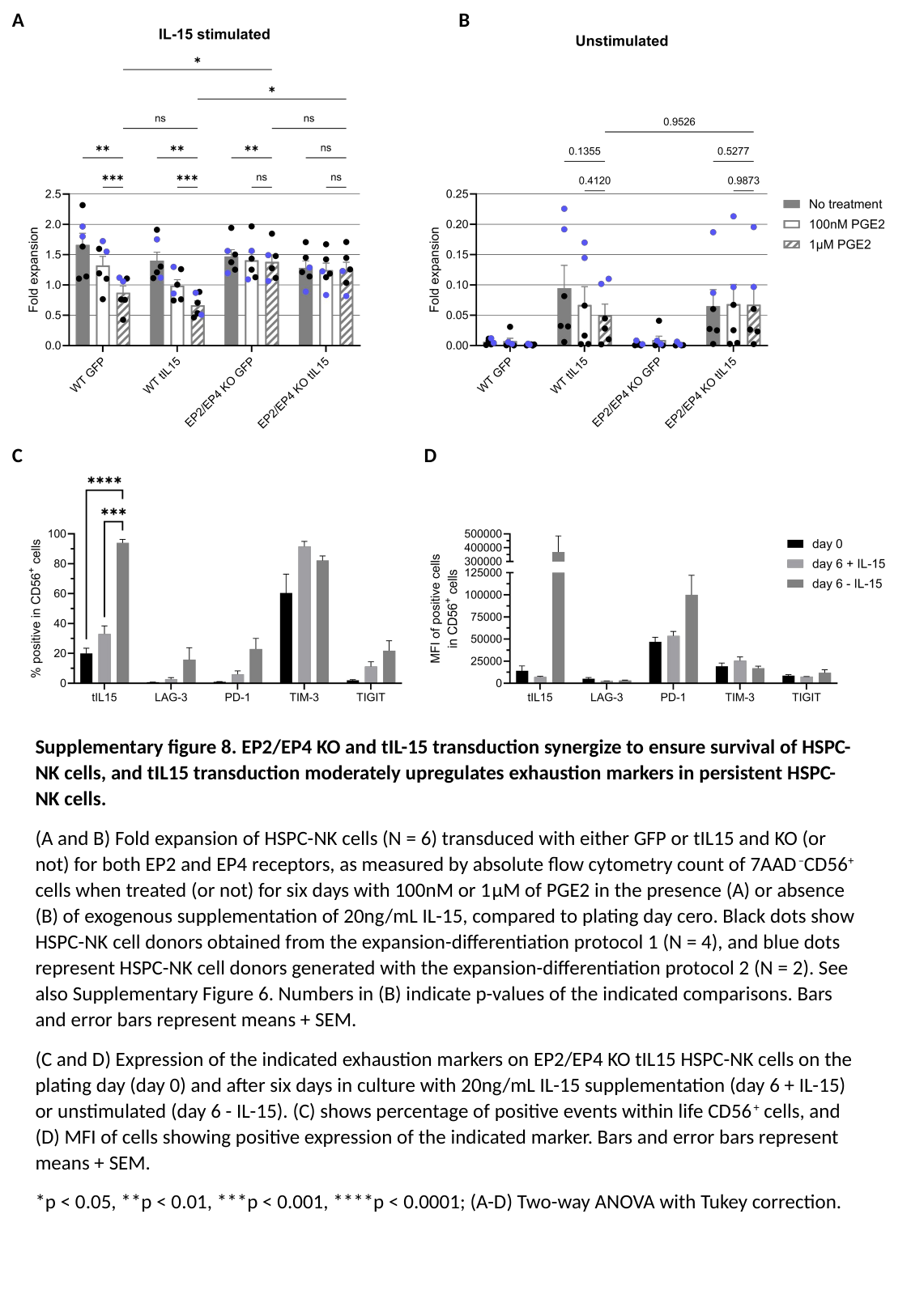

B
A
C
D
Supplementary figure 8. EP2/EP4 KO and tIL-15 transduction synergize to ensure survival of HSPC-NK cells, and tIL15 transduction moderately upregulates exhaustion markers in persistent HSPC-NK cells.
(A and B) Fold expansion of HSPC-NK cells (N = 6) transduced with either GFP or tIL15 and KO (or not) for both EP2 and EP4 receptors, as measured by absolute flow cytometry count of 7AAD–CD56+ cells when treated (or not) for six days with 100nM or 1μM of PGE2 in the presence (A) or absence (B) of exogenous supplementation of 20ng/mL IL-15, compared to plating day cero. Black dots show HSPC-NK cell donors obtained from the expansion-differentiation protocol 1 (N = 4), and blue dots represent HSPC-NK cell donors generated with the expansion-differentiation protocol 2 (N = 2). See also Supplementary Figure 6. Numbers in (B) indicate p-values of the indicated comparisons. Bars and error bars represent means + SEM.
(C and D) Expression of the indicated exhaustion markers on EP2/EP4 KO tIL15 HSPC-NK cells on the plating day (day 0) and after six days in culture with 20ng/mL IL-15 supplementation (day 6 + IL-15) or unstimulated (day 6 - IL-15). (C) shows percentage of positive events within life CD56+ cells, and (D) MFI of cells showing positive expression of the indicated marker. Bars and error bars represent means + SEM.
*p < 0.05, **p < 0.01, ***p < 0.001, ****p < 0.0001; (A-D) Two-way ANOVA with Tukey correction.

## Slide 12
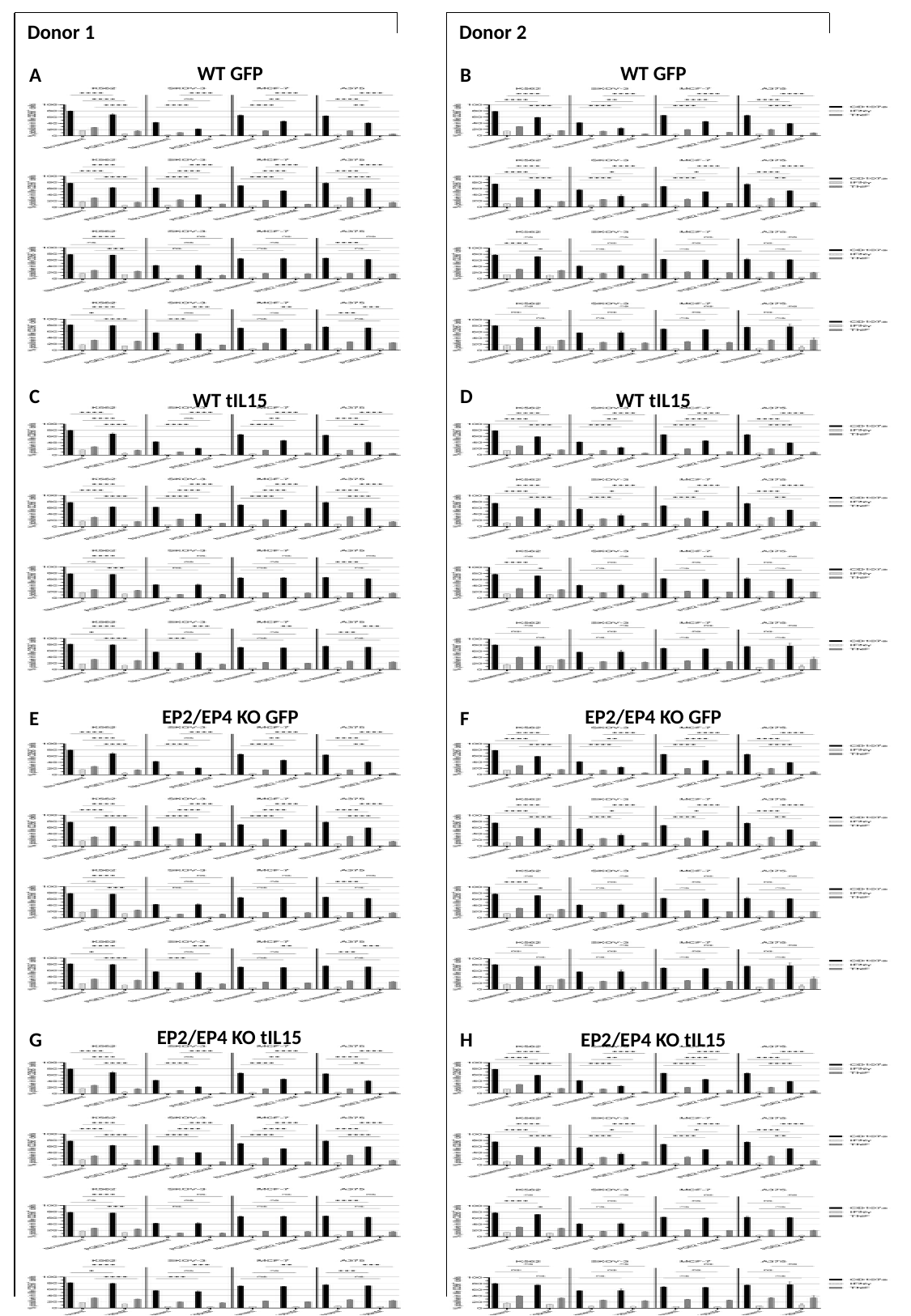

Donor 1
Donor 2
WT GFP
WT GFP
A
B
C
D
WT tIL15
WT tIL15
EP2/EP4 KO GFP
EP2/EP4 KO GFP
E
F
EP2/EP4 KO tIL15
EP2/EP4 KO tIL15
G
H

## Slide 13
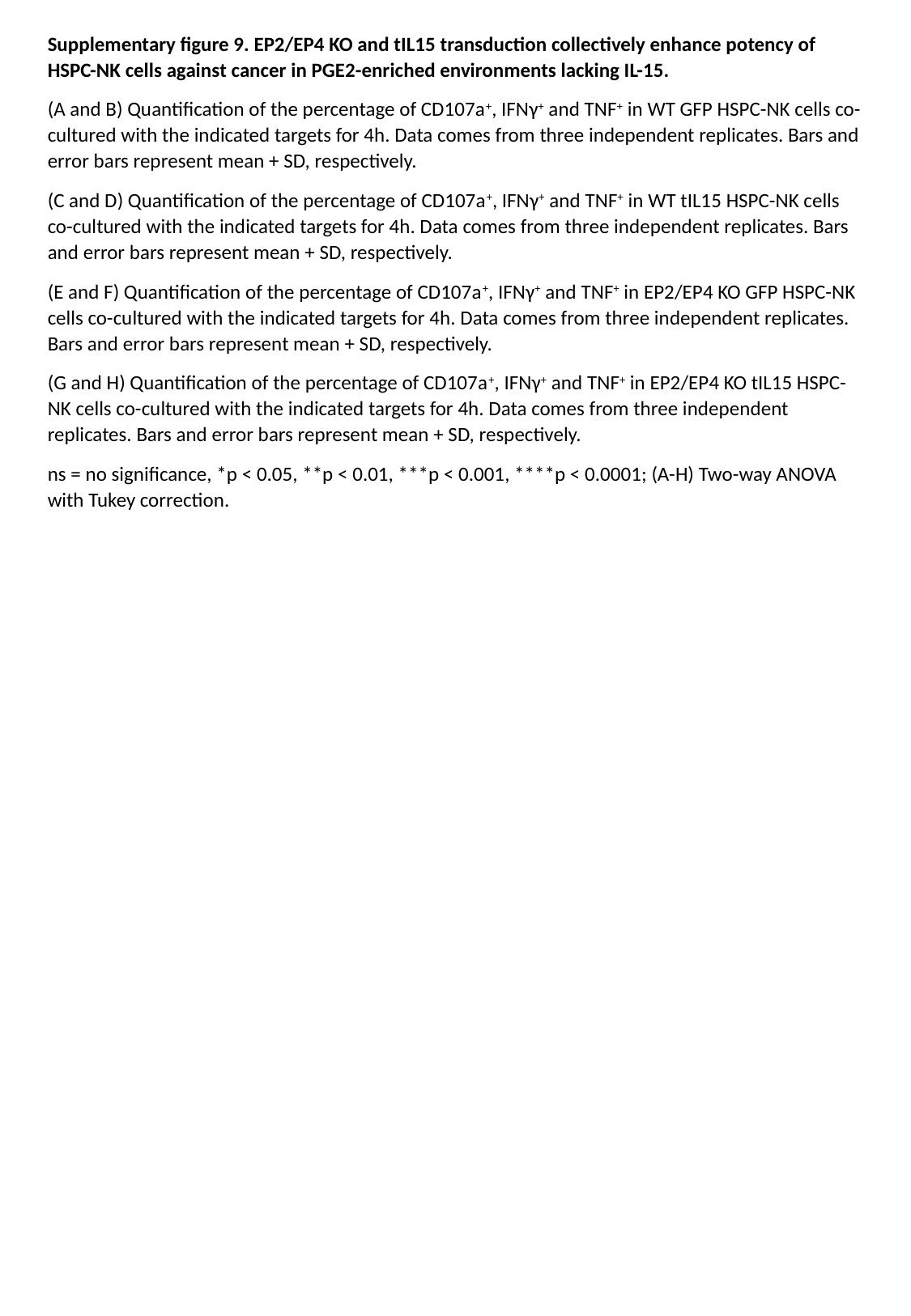

Supplementary figure 9. EP2/EP4 KO and tIL15 transduction collectively enhance potency of HSPC-NK cells against cancer in PGE2-enriched environments lacking IL-15.
(A and B) Quantification of the percentage of CD107a+, IFNγ+ and TNF+ in WT GFP HSPC-NK cells co-cultured with the indicated targets for 4h. Data comes from three independent replicates. Bars and error bars represent mean + SD, respectively.
(C and D) Quantification of the percentage of CD107a+, IFNγ+ and TNF+ in WT tIL15 HSPC-NK cells co-cultured with the indicated targets for 4h. Data comes from three independent replicates. Bars and error bars represent mean + SD, respectively.
(E and F) Quantification of the percentage of CD107a+, IFNγ+ and TNF+ in EP2/EP4 KO GFP HSPC-NK cells co-cultured with the indicated targets for 4h. Data comes from three independent replicates. Bars and error bars represent mean + SD, respectively.
(G and H) Quantification of the percentage of CD107a+, IFNγ+ and TNF+ in EP2/EP4 KO tIL15 HSPC-NK cells co-cultured with the indicated targets for 4h. Data comes from three independent replicates. Bars and error bars represent mean + SD, respectively.
ns = no significance, *p < 0.05, **p < 0.01, ***p < 0.001, ****p < 0.0001; (A-H) Two-way ANOVA with Tukey correction.

## Slide 14
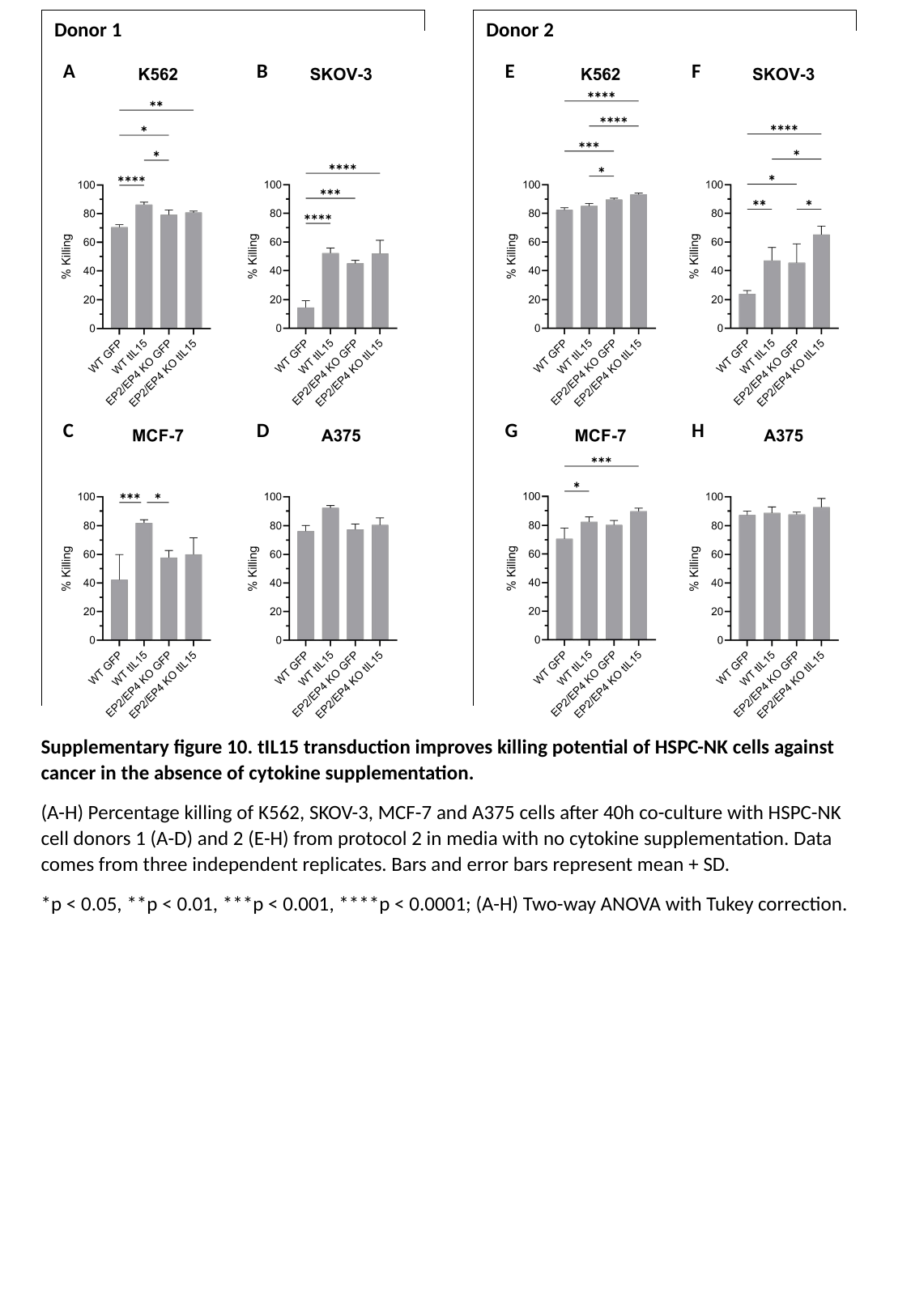

Donor 1
Donor 2
A
B
E
F
C
D
G
H
Supplementary figure 10. tIL15 transduction improves killing potential of HSPC-NK cells against cancer in the absence of cytokine supplementation.
(A-H) Percentage killing of K562, SKOV-3, MCF-7 and A375 cells after 40h co-culture with HSPC-NK cell donors 1 (A-D) and 2 (E-H) from protocol 2 in media with no cytokine supplementation. Data comes from three independent replicates. Bars and error bars represent mean + SD.
*p < 0.05, **p < 0.01, ***p < 0.001, ****p < 0.0001; (A-H) Two-way ANOVA with Tukey correction.

## Slide 15
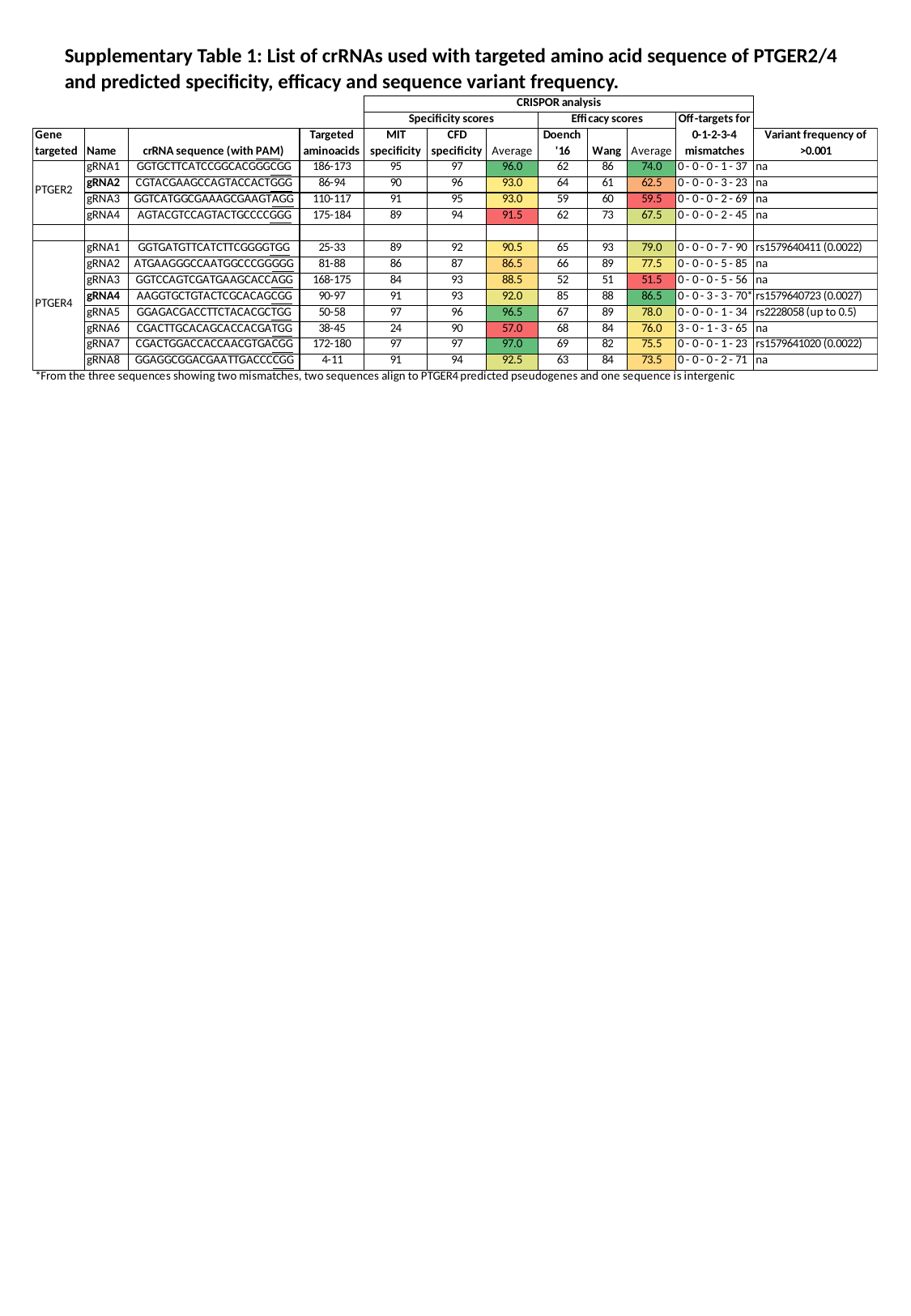

Supplementary Table 1: List of crRNAs used with targeted amino acid sequence of PTGER2/4 and predicted specificity, efficacy and sequence variant frequency.

## Slide 16
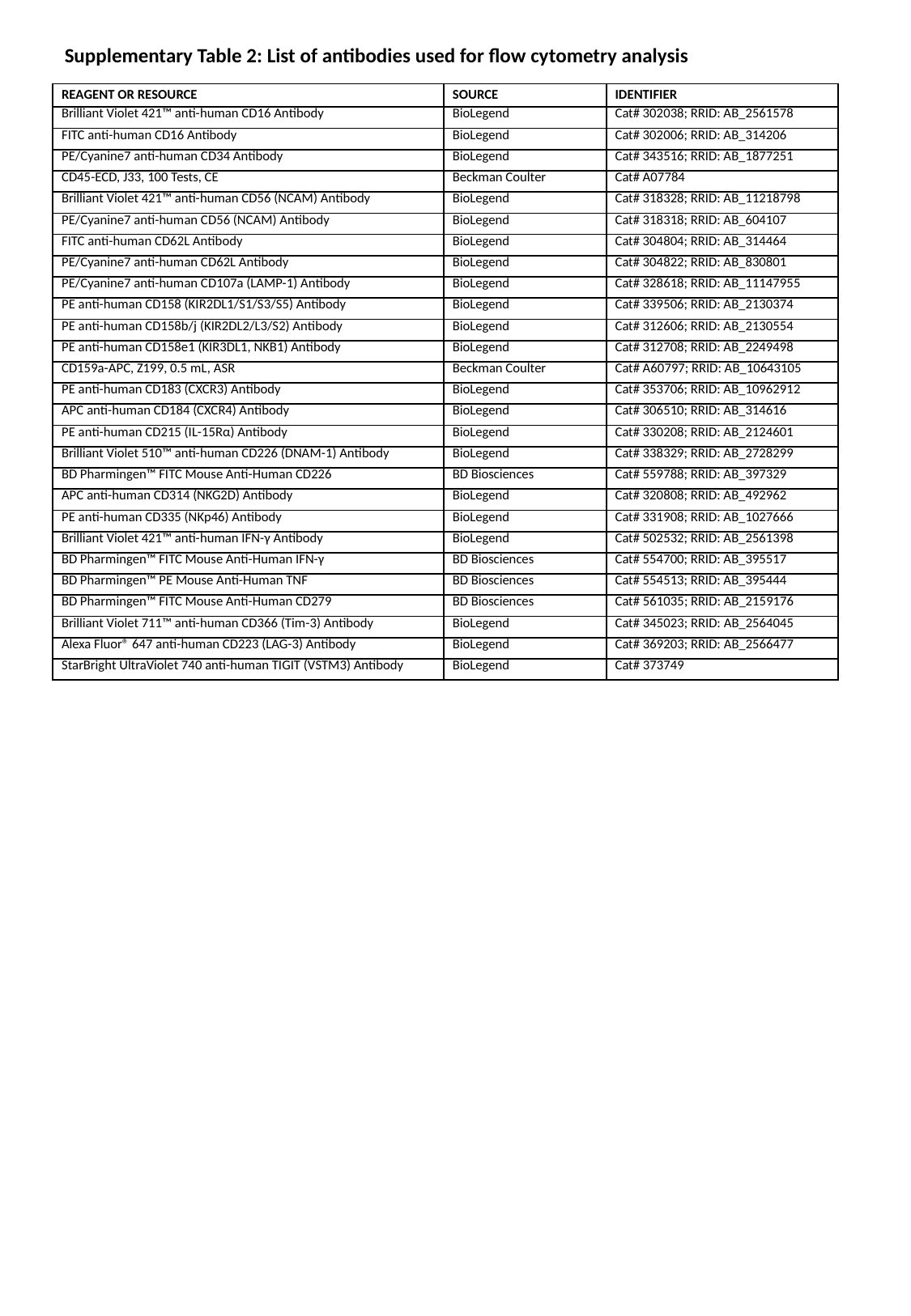

Supplementary Table 2: List of antibodies used for flow cytometry analysis
| REAGENT OR RESOURCE | SOURCE | IDENTIFIER |
| --- | --- | --- |
| Brilliant Violet 421™ anti-human CD16 Antibody | BioLegend | Cat# 302038; RRID: AB\_2561578 |
| FITC anti-human CD16 Antibody | BioLegend | Cat# 302006; RRID: AB\_314206 |
| PE/Cyanine7 anti-human CD34 Antibody | BioLegend | Cat# 343516; RRID: AB\_1877251 |
| CD45-ECD, J33, 100 Tests, CE | Beckman Coulter | Cat# A07784 |
| Brilliant Violet 421™ anti-human CD56 (NCAM) Antibody | BioLegend | Cat# 318328; RRID: AB\_11218798 |
| PE/Cyanine7 anti-human CD56 (NCAM) Antibody | BioLegend | Cat# 318318; RRID: AB\_604107 |
| FITC anti-human CD62L Antibody | BioLegend | Cat# 304804; RRID: AB\_314464 |
| PE/Cyanine7 anti-human CD62L Antibody | BioLegend | Cat# 304822; RRID: AB\_830801 |
| PE/Cyanine7 anti-human CD107a (LAMP-1) Antibody | BioLegend | Cat# 328618; RRID: AB\_11147955 |
| PE anti-human CD158 (KIR2DL1/S1/S3/S5) Antibody | BioLegend | Cat# 339506; RRID: AB\_2130374 |
| PE anti-human CD158b/j (KIR2DL2/L3/S2) Antibody | BioLegend | Cat# 312606; RRID: AB\_2130554 |
| PE anti-human CD158e1 (KIR3DL1, NKB1) Antibody | BioLegend | Cat# 312708; RRID: AB\_2249498 |
| CD159a-APC, Z199, 0.5 mL, ASR | Beckman Coulter | Cat# A60797; RRID: AB\_10643105 |
| PE anti-human CD183 (CXCR3) Antibody | BioLegend | Cat# 353706; RRID: AB\_10962912 |
| APC anti-human CD184 (CXCR4) Antibody | BioLegend | Cat# 306510; RRID: AB\_314616 |
| PE anti-human CD215 (IL-15Rα) Antibody | BioLegend | Cat# 330208; RRID: AB\_2124601 |
| Brilliant Violet 510™ anti-human CD226 (DNAM-1) Antibody | BioLegend | Cat# 338329; RRID: AB\_2728299 |
| BD Pharmingen™ FITC Mouse Anti-Human CD226 | BD Biosciences | Cat# 559788; RRID: AB\_397329 |
| APC anti-human CD314 (NKG2D) Antibody | BioLegend | Cat# 320808; RRID: AB\_492962 |
| PE anti-human CD335 (NKp46) Antibody | BioLegend | Cat# 331908; RRID: AB\_1027666 |
| Brilliant Violet 421™ anti-human IFN-γ Antibody | BioLegend | Cat# 502532; RRID: AB\_2561398 |
| BD Pharmingen™ FITC Mouse Anti-Human IFN-γ | BD Biosciences | Cat# 554700; RRID: AB\_395517 |
| BD Pharmingen™ PE Mouse Anti-Human TNF | BD Biosciences | Cat# 554513; RRID: AB\_395444 |
| BD Pharmingen™ FITC Mouse Anti-Human CD279 | BD Biosciences | Cat# 561035; RRID: AB\_2159176 |
| Brilliant Violet 711™ anti-human CD366 (Tim-3) Antibody | BioLegend | Cat# 345023; RRID: AB\_2564045 |
| Alexa Fluor® 647 anti-human CD223 (LAG-3) Antibody | BioLegend | Cat# 369203; RRID: AB\_2566477 |
| StarBright UltraViolet 740 anti-human TIGIT (VSTM3) Antibody | BioLegend | Cat# 373749 |

## Slide 17
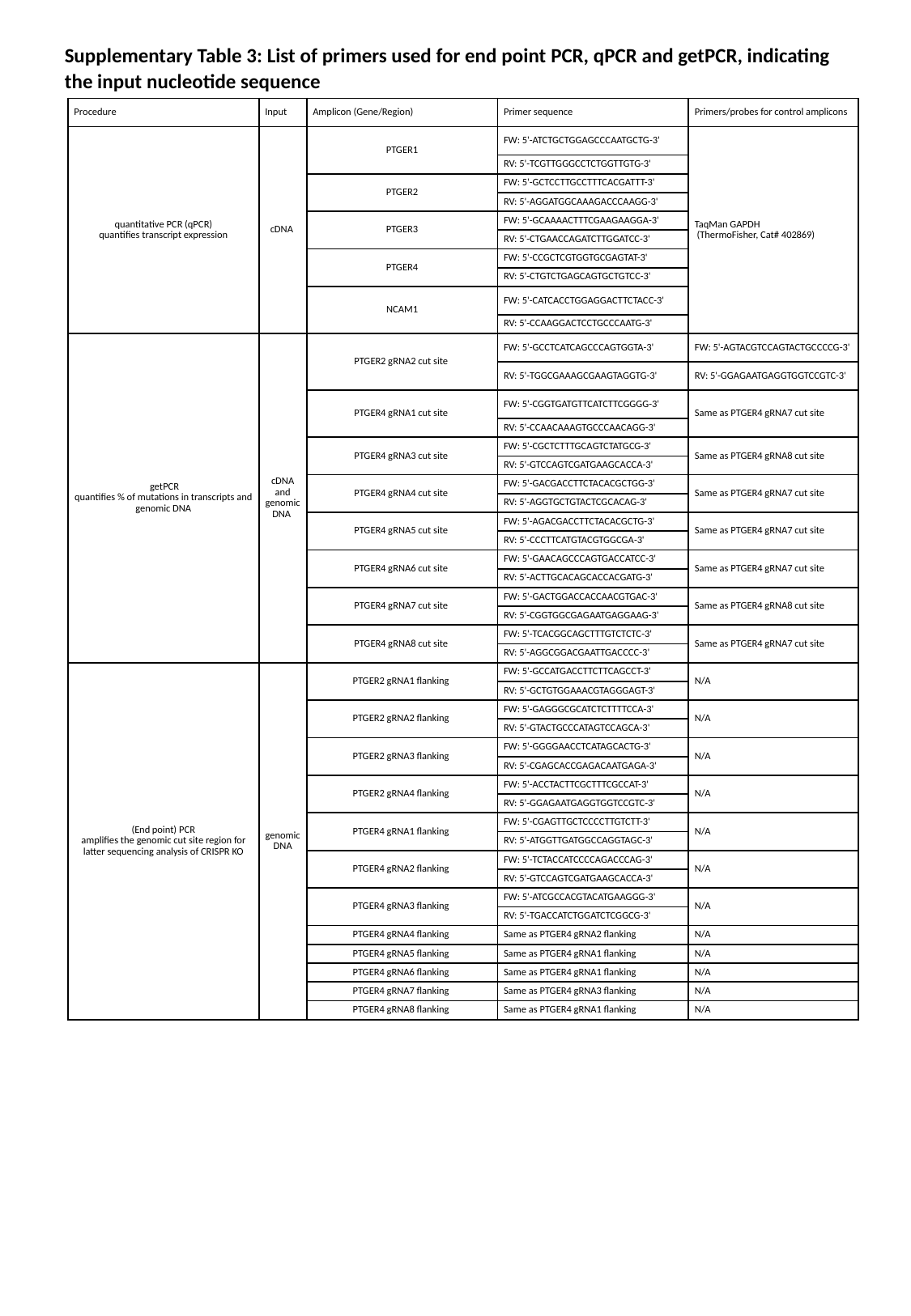

Supplementary Table 3: List of primers used for end point PCR, qPCR and getPCR, indicating the input nucleotide sequence
| Procedure | Input | Amplicon (Gene/Region) | Primer sequence | Primers/probes for control amplicons |
| --- | --- | --- | --- | --- |
| quantitative PCR (qPCR) quantifies transcript expression | cDNA | PTGER1 | FW: 5'-ATCTGCTGGAGCCCAATGCTG-3' | TaqMan GAPDH (ThermoFisher, Cat# 402869) |
| | | | RV: 5'-TCGTTGGGCCTCTGGTTGTG-3' | |
| | | PTGER2 | FW: 5'-GCTCCTTGCCTTTCACGATTT-3' | |
| | | | RV: 5'-AGGATGGCAAAGACCCAAGG-3' | |
| | | PTGER3 | FW: 5'-GCAAAACTTTCGAAGAAGGA-3' | |
| | | | RV: 5'-CTGAACCAGATCTTGGATCC-3' | |
| | | PTGER4 | FW: 5'-CCGCTCGTGGTGCGAGTAT-3' | |
| | | | RV: 5'-CTGTCTGAGCAGTGCTGTCC-3' | |
| | | NCAM1 | FW: 5'-CATCACCTGGAGGACTTCTACC-3' | |
| | | | RV: 5'-CCAAGGACTCCTGCCCAATG-3' | |
| getPCR quantifies % of mutations in transcripts and genomic DNA | cDNA and genomic DNA | PTGER2 gRNA2 cut site | FW: 5'-GCCTCATCAGCCCAGTGGTA-3' | FW: 5'-AGTACGTCCAGTACTGCCCCG-3' |
| | | | RV: 5'-TGGCGAAAGCGAAGTAGGTG-3' | RV: 5'-GGAGAATGAGGTGGTCCGTC-3' |
| | | PTGER4 gRNA1 cut site | FW: 5'-CGGTGATGTTCATCTTCGGGG-3' | Same as PTGER4 gRNA7 cut site |
| | | | RV: 5'-CCAACAAAGTGCCCAACAGG-3' | |
| | | PTGER4 gRNA3 cut site | FW: 5'-CGCTCTTTGCAGTCTATGCG-3' | Same as PTGER4 gRNA8 cut site |
| | | | RV: 5'-GTCCAGTCGATGAAGCACCA-3' | |
| | | PTGER4 gRNA4 cut site | FW: 5'-GACGACCTTCTACACGCTGG-3' | Same as PTGER4 gRNA7 cut site |
| | | | RV: 5'-AGGTGCTGTACTCGCACAG-3' | |
| | | PTGER4 gRNA5 cut site | FW: 5'-AGACGACCTTCTACACGCTG-3' | Same as PTGER4 gRNA7 cut site |
| | | | RV: 5'-CCCTTCATGTACGTGGCGA-3' | |
| | | PTGER4 gRNA6 cut site | FW: 5'-GAACAGCCCAGTGACCATCC-3' | Same as PTGER4 gRNA7 cut site |
| | | | RV: 5'-ACTTGCACAGCACCACGATG-3' | |
| | | PTGER4 gRNA7 cut site | FW: 5'-GACTGGACCACCAACGTGAC-3' | Same as PTGER4 gRNA8 cut site |
| | | | RV: 5'-CGGTGGCGAGAATGAGGAAG-3' | |
| | | PTGER4 gRNA8 cut site | FW: 5'-TCACGGCAGCTTTGTCTCTC-3' | Same as PTGER4 gRNA7 cut site |
| | | | RV: 5'-AGGCGGACGAATTGACCCC-3' | |
| (End point) PCR amplifies the genomic cut site region for latter sequencing analysis of CRISPR KO | genomic DNA | PTGER2 gRNA1 flanking | FW: 5'-GCCATGACCTTCTTCAGCCT-3' | N/A |
| | | | RV: 5'-GCTGTGGAAACGTAGGGAGT-3' | |
| | | PTGER2 gRNA2 flanking | FW: 5'-GAGGGCGCATCTCTTTTCCA-3' | N/A |
| | | | RV: 5'-GTACTGCCCATAGTCCAGCA-3' | |
| | | PTGER2 gRNA3 flanking | FW: 5'-GGGGAACCTCATAGCACTG-3' | N/A |
| | | | RV: 5'-CGAGCACCGAGACAATGAGA-3' | |
| | | PTGER2 gRNA4 flanking | FW: 5'-ACCTACTTCGCTTTCGCCAT-3' | N/A |
| | | | RV: 5'-GGAGAATGAGGTGGTCCGTC-3' | |
| | | PTGER4 gRNA1 flanking | FW: 5'-CGAGTTGCTCCCCTTGTCTT-3' | N/A |
| | | | RV: 5'-ATGGTTGATGGCCAGGTAGC-3' | |
| | | PTGER4 gRNA2 flanking | FW: 5'-TCTACCATCCCCAGACCCAG-3' | N/A |
| | | | RV: 5'-GTCCAGTCGATGAAGCACCA-3' | |
| | | PTGER4 gRNA3 flanking | FW: 5'-ATCGCCACGTACATGAAGGG-3' | N/A |
| | | | RV: 5'-TGACCATCTGGATCTCGGCG-3' | |
| | | PTGER4 gRNA4 flanking | Same as PTGER4 gRNA2 flanking | N/A |
| | | PTGER4 gRNA5 flanking | Same as PTGER4 gRNA1 flanking | N/A |
| | | PTGER4 gRNA6 flanking | Same as PTGER4 gRNA1 flanking | N/A |
| | | PTGER4 gRNA7 flanking | Same as PTGER4 gRNA3 flanking | N/A |
| | | PTGER4 gRNA8 flanking | Same as PTGER4 gRNA1 flanking | N/A |
